# Hydrogels for Cancer Immunotherapy: Strategies From Construction to Application

**DOI:** 10.1002/mco2.70615

**Published:** 2026-02-02

**Authors:** Xiaohua Chen, Shan Wu, Yueyang Zhang, Lilu Feng, Yang Chen, Xuzhao Yang, Ke Men, Jiuqun Zhu, Ming Li

**Affiliations:** ^1^ Department of Pharmacy Personalized Drug Therapy Key Laboratory of Sichuan Province Sichuan Academy of Medical Sciences & Sichuan Provincial People's Hospital School of Medicine University of Electronic Science and Technology of China Chengdu Sichuan Province China; ^2^ State Key Laboratory of Biotherapy and Cancer Center West China Hospital of Sichuan University Chengdu Sichuan Province China; ^3^ Department of Cardiovascular Sichuan Provincial People's Hospital University of Electronic Science and Technology of China Chengdu Sichuan Province China

**Keywords:** biomaterial, cancer, hydrogel, immunotherapy

## Abstract

Cancer is a complex disease characterized by systemic dysfunction, necessitating a balance between therapeutic efficacy and safety. Immunotherapy is a core treatment approach for activating the antitumor immune response in the human body. The development of intelligent hydrogels has provided an innovative platform for tumor immunotherapy, owing to their adjustable properties for controlled drug delivery and immune modulation. Tumor immunotherapy has achieved remarkable success in recent years. However, it continues to face critical challenges such as targeting and delivery barriers, suppression by the TME, and immune evasion and drug resistance. In response, as injectable or implantable biomaterials, hydrogels are emerging as a promising platform to address these limitations by enabling localized, controllable drug delivery and immunomodulation. This review systematically categorizes contemporary hydrogel construction strategies tailored for immunotherapy, highlighting the distinct advantages of specific architectures in diverse clinical contexts. By classifying hydrogel applications according to immune‐based strategies, the work underscores their multifunctional utility as precision delivery platforms and modulators of the immune microenvironment. This comprehensive overview elucidates the progress and design principles of hydrogel‐based immunotherapeutic platforms, providing valuable insights to guide future research and development in this evolving field.

## Graphical abstract

Hydrogels offer an innovative platform for cancer immunotherapy. Through tailorable crosslinking strategies, they form customizable drug delivery platforms that address diverse therapeutic requirements by loading specific immunotherapeutic agents. These systems demonstrate significant clinical potential for precise tumor immunotherapy.

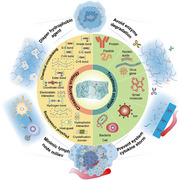

## 1 Introduction

Cancer biotherapy has emerged as the fourth major treatment modality following surgery, radiotherapy, and chemotherapy [1, 2]. It works by activating the patient's immune system or using biological mechanisms to precisely target and eliminate tumor cells, offering a safer and more personalized therapeutic approach. However, cancer immunotherapy still faces challenges such as poor tumor accumulation, limited drug synergy, and insufficient biocompatibility [3, 4, 5, 6]. These issues highlight the need for improved drug delivery systems.

Hydrogels represent a promising solution due to their tunable physical properties, porous structure, and biocompatibility. They can be designed to respond to stimuli like pH, temperature, or enzymes, enabling controlled drug release [7, 8]. Recent studies have demonstrated the potential of smart hydrogels to enhance therapeutic efficacy in tumor models by reprogramming the immune microenvironment, enabling precise recruitment and activation of immune cells, and synergizing localized immunomodulation with chemotherapy, photothermal therapy (PTT), and other treatment modalities [9, 10, 11, 12]. These achievements highlight the significant progress of hydrogels in the field of tumor immunotherapy. Nevertheless, despite these pronounced advantages, the clinical translation of hydrogel technology continues to encounter multiple challenges. These include balancing biocompatibility with long‐term safety, overcoming process bottlenecks in scaling up from laboratory preparation to industrial production, and accurately predicting hydrogel behavior within complex in vivo environments [13, 14, 15]. Although existing reviews have systematically summarized the physicochemical properties of hydrogels and their applications in drug delivery, they often overemphasize the material's delivery function while overlooking its role as an immunomodulatory platform that enhances therapeutic outcomes through interaction with the tumor‐associated immune system. Moreover, there remains a lack of in‐depth discussion on the mechanisms underlying hydrogel‐immune system interactions.

**FIGURE 1:**
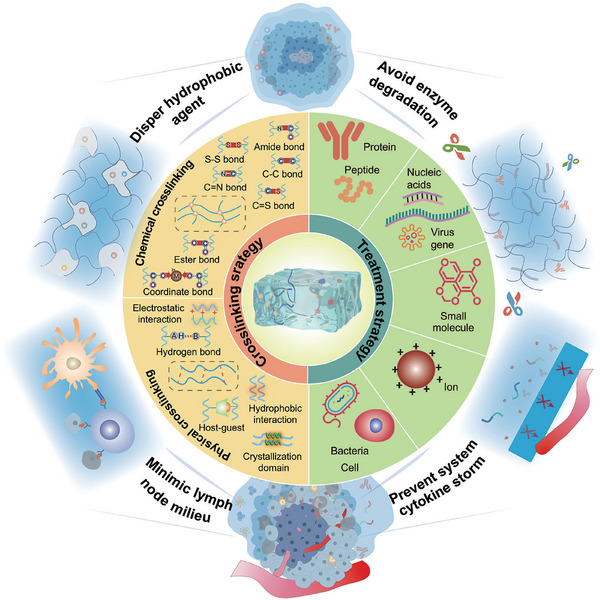
Schematic overview of hydrogel‐based platforms for tumor immunotherapy. The diagram categorizes systems according to their construction strategies, including chemical, photocurable, thermal, intermolecular, and biochemical. And their application strategies, such as protein/peptide, gene, small molecule, ion, and bacterial/cellular. The illustration highlights the integration of material design with immunotherapeutic function for localized and sustained antitumor responses.

Therefore, this review aims to systematically consolidate the latest advances in hydrogels for tumor immunotherapy. It focuses particularly on the profound impact of hydrogel crosslinking strategies on immune regulation and explores advanced design principles for multimechanistic synergistic therapy rooted in immunologically relevant engineering. Furthermore, the review specifically summarizes application experiences and therapeutic outcomes from hydrogel platforms that have entered clinical stages or have been commercialized, with the goal of providing a theoretical foundation and directional guidance for future research (Figure 1).

## 2 Construction Strategy

In the realm of tumor immunotherapy, hydrogels have emerged as versatile platforms for multimodal drug delivery systems, leveraging their exceptional biocompatibility and tunable physicochemical properties. These intelligent materials can be engineered through diverse fabrication strategies. Based on the chemical nature of crosslinking bonds, the construction strategies of hydrogels can be classified into chemical crosslinking and physical crosslinking: chemical crosslinking relies on irreversible covalent bonds, while physical crosslinking depends on reversible noncovalent interactions. The permanence of covalent bonds and the dynamic reversibility of noncovalent bonds determine the mechanical behavior and response characteristics of hydrogels, each fabrication approach imparts distinct functional attributes [16]. This review systematically categorizes and elaborates on contemporary hydrogel construction strategies tailored for immunotherapeutic applications, emphasizing the advantages of specific architectures in particular circumstances. These construction strategies are summarized in Table 1.

**TABLE 1:** Hydrogel crosslinking properties.

Bond type	Crosslinking strategy	Drug loading capacity	Mechanical strength	Degradation kinetics	Release half‐life	Key stimuli	Immunocompatibility	Key characteristics	Main weaknesses	Clinical relevance potential
Chemical crosslinking	C‐C bond	Medium‐high	Very high	Very slow, essentially nondegradable	Weeks to months	None	Medium (residual monomers/initiators may be toxic)	High stability, tunable stiffness	Lack of bio‐responsiveness, degradation products may cause inflammation.	For implants requiring long‐term structural support
	C‐S bond	High	Medium–high	Slow, can be designed for enzyme response	Days to weeks	Enzymes	High (mild reaction conditions)	High selectivity, high reaction efficiency	Relatively weak bond energy, stability inferior to C‐C bond	For controlled drug delivery
	S‐S bond	Medium‐high	Medium	Fast, GSH	Hours to days	GSH	High	Self‐healing, shape memory, redox‐responsive	May degrade too rapidly in highly reductive environments	For smart, responsive drug release
	C = N bond	Medium	Medium	pH‐responsive	Hours to days	Acidic pH	High (bioorthogonal, water byproduct)	Tunable gelation time/properties, pH‐responsive	Imines may be unstable in acidic TME.	For TME‐targeted delivery
	Ester bond	Medium	Medium/high	Hydrolytic/ROS‐responsive	Days	ROS	Medium/high (depends on precursors)	ROS‐responsive, fast formation	Limited stability in body fluids or high ROS environments	For ROS‐related combination therapies
	Amide bond	Low‐medium	High	Very slow, enzymatically stable, acid‐stable	Weeks	None	High	High stability, promotes cell adhesion	Lack of responsiveness, difficult to degrade	For long‐term implants and tissue adhesion
	Coordinate bond	Variable	Variable (low–high)	Ion/pH/temperature responsive	Hours to weeks	pH, temperature, Ions	Medium (depends on metal ion)	Self‐healing, thermal/chemical responsive, can load drugs	Metal ions may be toxic	For stimuli‐responsive and self‐healing systems
Physical crosslinking	Electrostatic interaction	High	Low/medium	Ion strength/pH responsive	Days to weeks	Ionic strength, pH	High	Self‐healing, shear‐thinning, smart release	Low mechanical strength, sensitive to physiological environment	For injectable and environmentally responsive formulations
	Hydrogen bond	Medium	Low/medium	Reversible, temperature/pH responsive	Days	Temperature, pH	Very high	Excellent self‐healing and injectability, programmable (DNA)	Generally low mechanical strength	For cell delivery, vaccines, and injectable platforms
	Hydrophobic interaction	High for hydrophobic drugs	Low/medium	Temperature responsive (thermogelling)	Days to weeks	Temperature	High	Thermoresponsive, can form micelles to load hydrophobic drugs	May disintegrate due to dilution in vivo	For thermally induced in situ gelation and drug delivery
	Crystallization domain	Low‐medium	Very high	Very slow, stable	Weeks	None	High	High toughness and strength, antiswelling	Slow formation process, lack of responsiveness	For scaffolds requiring high mechanical strength
	Host–guest	High	Low/medium	Dynamic, can be designed for stimulus response	Days to weeks	Shear, light, competitor molecules	Very high	Excellent self‐healing and injectability, functionalizable	Low mechanical strength, loading capacity may be limited	For advanced drug and cell delivery systems

The clinical trials data sources: https://clinicaltrials.gov/.

### 2.1 Chemical Crosslinking

Chemical crosslinking connects polymer chains through covalent bonds to form a permanent three‐dimensional network. Its most prominent characteristics are high structural stability and mechanical strength. Owing to the strong bond energy of covalent linkages, the network structure of chemically crosslinked hydrogels is typically irreversible, enabling them to maintain their shape over extended periods in aqueous or solvent environments without dissolving. Furthermore, these hydrogels are capable of withstanding substantial mechanical stresses. Based on these attributes, chemically crosslinked hydrogels are widely utilized in fields that demand long‐term stability and structural integrity.

#### 2.1.1 C─C Bond

C─C bonds form the fundamental structural framework of organic molecules. In hydrogel synthesis, C─C bonds can be established between precursor molecules via free radical reactions without introducing additional atoms. These bonds exhibit inherent nonpolarity, thermodynamic stability, dynamic inertia, and high rigidity. Hydrogels incorporating C─C bonds typically demonstrate enhanced stability and prolonged degradation rates [17]. For instance, Sun et al. [18] developed a durable, long‐acting hydrogel by modifying hyaluronic acid with glycidyl methacrylate to form C─C bonds between methacrylate groups and N‐isopropyl acrylamide (NIPAM). Post‐bond formation, the hyaluronic acid‐based hydrogel exhibited minimal degradation over 16 days in 5 U/ml hyaluronidase. Furthermore, the mechanical strength of the hydrogel was not only improved but also tunable through adjustments in crosslinking density. Lee et al. [19] synthesized a hydrogel by conjugating hydroxyphenyl propionic acid‐modified gelatin (GHPA) via C─C bonds generated by free radicals from the enzymatic reaction between H_2_O_2_ and horseradish peroxidase. Increasing the feed ratios of GHPA and H_2_O_2_ yielded storage moduli ranging from ∼100 to ∼2500 Pa, reflecting enhanced crosslinking. This tunable mechanical property enabled broad applications in gene therapy, targeted therapy, and cell therapy.

In hydrogel preparation, C─C bonds are typically formed through reactions between (meth)acrylate‐conjugated polymers. As precursors, these polymers utilize molecular double bonds to facilitate repeated addition reactions, thereby constructing three‐dimensional hydrogel networks. Seo et al. [20] introduced methacrylated glycol groups onto chitosan (CS) via nucleophilic substitution between glycidyl methacrylate and CS. The vinyl groups in the methacrylated glycol moiety facilitated gelation through C─C bond formation via free radical reactions. This strategy enabled the synthesis of C─C bond‐based hydrogels, which have since been widely adopted in subsequent studies. Meenach et al. [21] modified polyethylene glycol (PEG) with methyl ether methacrylate and methyl dimethacrylate. C─C crosslinking between the vinyl groups of the modified moieties formed a three‐dimensional hydrogel network, imparting high compressive strength and structural stability. C─C bond hydrogels, crosslinked via covalent bonds between (meth)acrylate‐conjugated polymers, are extensively utilized in hydrogel fabrication due to their robust mechanical properties. Che et al. [22] engineered a semi‐interpenetrating polymer network (semi‐IPN) with C─C bonds, achieving enhanced mechanical performance. Du et al. [23] fabricated a hydrogel through free radical polymerization of carbon‐carbon double bonds, resulting in nonswelling behavior and superior mechanical strength. C─C bond‐crosslinked hydrogels are characterized by their exceptional mechanical strength and extremely slow degradation rate, thereby rendering them highly suitable for applications requiring long‐term structural support and sustained drug release. However, their lack of biological responsiveness and the potential cytotoxicity associated with unreacted monomers limit their utility in applications that demand smart, on‐demand drug delivery or codelivery with living cells. These hydrogels are more appropriate for the encapsulation of stable signaling molecules to achieve prolonged immune modulation, rather than for the delivery of checkpoint inhibitors that require rapid, environmentally triggered release.

#### 2.1.2 C‐S Bond

**FIGURE 2:**
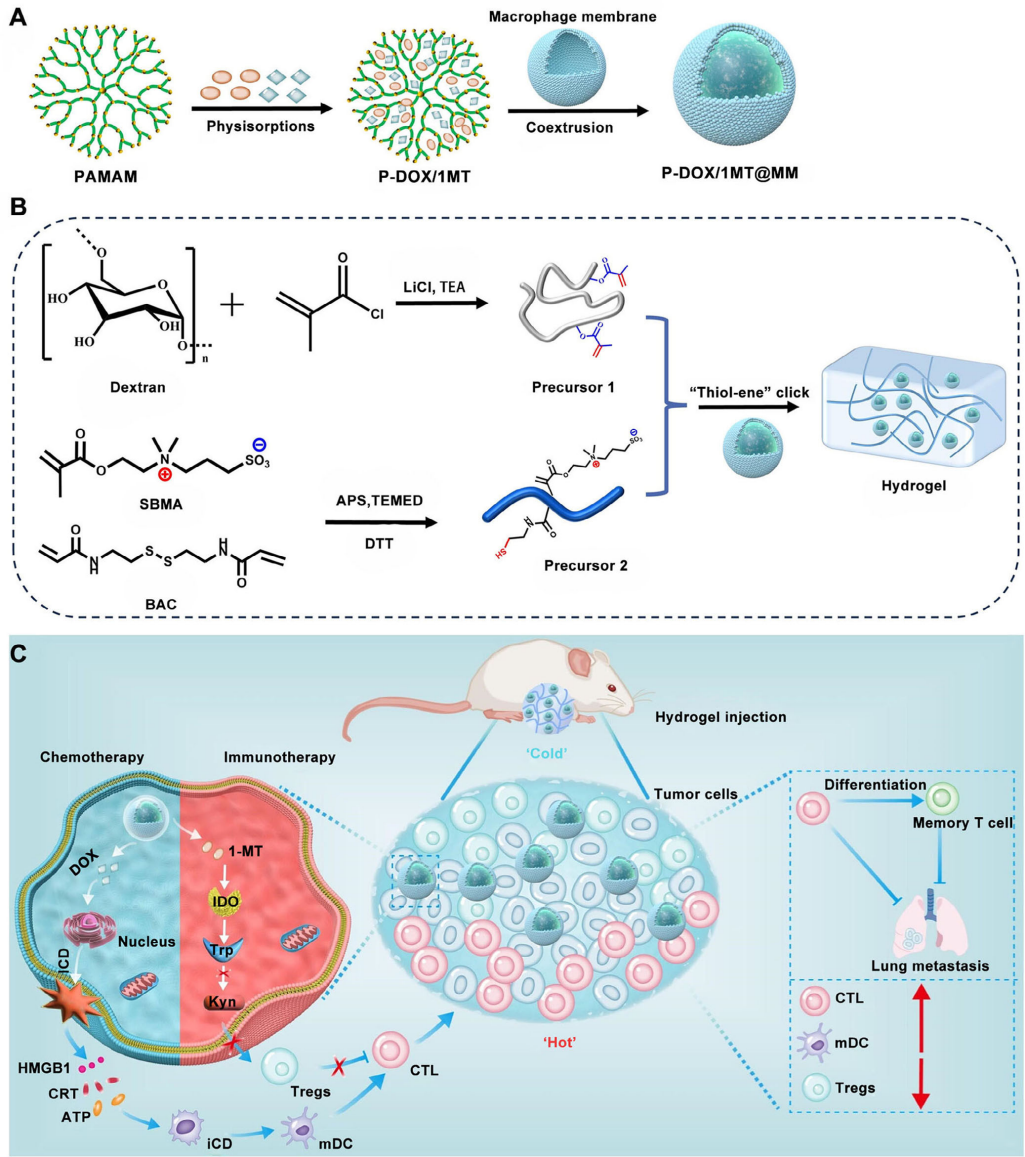
Schematic illustration of the P‐DOX/1MT@MM‐Gel for preventing postoperative tumor recurrence and lung metastasis: (A) Fabrication of P‐DOX/1MT@MM. The preparation of PAMAM dendrimer coloaded with DOX and 1‐MT, followed by macrophage membrane coating to form targeted unimicelles. (B) Synthesis of the injectable, zwitterionic hydrogel via “thiol‐ene” click chemistry. The preparation of the two hydrogel precursors and their rapid, in situ crosslinking upon mixing to form a biocompatible, shear‐thinning hydrogel network serving as a localized drug depot. (C) Sequential mechanism of action for inhibiting postoperative recurrence/metastasis and activating systemic antitumor immunity. Reprinted with permission from Ref. [30], Copyright 2024 American Chemical Society.

C‐S bonds are commonly synthesized via thiol‐Michael addition reactions. Due to sulfur's larger atomic radius and weaker covalent bond strength, C‐S bonds exhibit lower stability and higher reactivity compared with C─C bonds [24]. However, C‐S bonds offer distinct advantages: (1) facile bonding, (2) simple and efficient reaction pathways, and (3) high selectivity [25, 26, 27]. The thiol‐Michael addition reaction demonstrates high functional group conversion rates and selectivity, enabling the design of hydrogel networks with defined molecular architectures and tailored biological functions [28, 29]. Wang et al. [30] synthesized two precursors containing ‐SH and ‐ene groups, which were combined to trigger a thiol‐maleimide reaction, forming C‐S bonds. The resultant hydrogel exhibited low cytotoxicity, with hemolysis rates below 5%. Its porous structure conferred excellent drug loading and diffusion capabilities. This approach achieved high selectivity, yielding a hydrogel with a specific molecular structure and a spatially porous architecture (Figure 2). Additional studies have demonstrated that crosslinkers enhance the reactivity and selectivity of C‐S bond formation. Livingston et al. [31] prepared a thiol‐modified hyaluronic acid (SH–HA)‐based hydrogel using PEG diacrylate (PEGDA) as a crosslinker. The SH–HA and PEGDA reacted via ‐SH and ‐ene groups to form C‐S bonds, with a hydrogel forming within 1 h at a 4:1 precursor ratio. Ran et al. [32] engineered a rapid in situ‐forming PEG‐based hydrogel by designing multiarm PEG modified with thiol and maleimide groups. The Michael addition reaction accelerated with increasing functional group density, with multiarm PEG outperforming 4‐arm or 2‐arm PEG in crosslinking efficiency. When injected into the vitreous cavity, the hydrogel formed within <1 min due to high reactivity, remaining transparent and nonbiodegradable in rabbit eyes for 6 months. Li et al. [33] designed a collagenase‐responsive hydrogel using a 4‐arm PEG maleimide (4‐Arm‐PEG‐Mal), an N‐terminal maleimidated antimicrobial peptide (Mal‐AMP), and a collagenase‐cleavable peptide with two thiol groups (HS–VPM–SH). The hydrogel formed rapidly in situ via maleimide‐thiol bonding, with collagenase overexpression triggering controlled AMP release through VPM peptide hydrolysis. Zhuang et al. [34] synthesized C‐S bond hydrogels via Michael addition between polydopamine and thiohyaluronic acid (HA–SH), self‐assembling at 37°C. Yan et al. [35] prepared hydrogel prepolymers by dissolving Mal‐PEG with mercaptopeptides. The C‐S bond offers notable advantages, including mild reaction conditions, high efficiency, and bioorthogonality, along with minimal toxicity to cells and proteins. These properties make it particularly suitable for encapsulating bioactive molecules sensitive to shear stress or chemical environments, such as protein‐based drugs, antibodies, and even living cells. However, the relatively low bond energy of C‐S bonds generally results in hydrogels with inferior mechanical strength, and their structural stability in long‐term implantation may be compromised by slow hydrolysis. Therefore, in immunotherapy, C‐S crosslinked hydrogels are well suited for scenarios requiring rapid encapsulation and release of therapeutic antibodies or cytokines.

#### 2.1.3 S‐S Bond

Disulfide bonds are typically formed through the coupling of two thiol groups. Although covalent in nature, disulfide bonds exhibit dynamic characteristics, being susceptible to cleavage via reduction and reformation through oxidation [36]. In recent years, disulfide‐bond crosslinking has emerged as a widely adopted strategy for constructing hydrogels with porous, dynamic networks [37, 38]. Hydrogels crosslinked by disulfide bonds offer several advantages.

First, the reversible nature of disulfide bond formation enables the preparation of self‐healing hydrogels. Yang et al. [39] synthesized a self‐adhesive hydrogel using the natural compound thioctic acid (TA) and dopamine‐grafted cellulose nanofibers via a straightforward one‐step synthesis method. The hydrogel demonstrated robust self‐healing capabilities after repeated mechanical damage, attributed to the abundance of dynamic disulfide bonds in its network, which facilitate molecular chain dissociation and reorganization. This dynamic reversibility of disulfide bonds endows the hydrogels with exceptional self‐healing properties. Building on this characteristic, Ling et al. [40] reported that disulfide‐bond crosslinked hydrogels also exhibit shape‐memory behavior. By combining polyvinyl alcohol (PVA) with l‐cysteine (Cys), they designed a hydrogel with both shape‐memory and self‐healing functionalities. The hydrogel was fabricated by oxidizing sulfhydryl groups on the Cys side chains into disulfide bonds. Stress–strain tests confirmed that the hydrogel could self‐heal after thermal stimulation under ambient conditions. Furthermore, due to the redox responsiveness of disulfide bonds, the hydrogel displayed shape‐memory properties under redox stimuli. Second, disulfide bonds can be cleaved via reduction reactions, enabling redox‐responsive degradation behavior in hydrogels. Chen et al. [41] developed a keratin‐based hydrogel through a mercaptan–disulfide bond exchange strategy. Keratin, naturally rich in intramolecular disulfide bonds and free sulfhydryl groups, allowed the formation of intermolecular disulfide bonds via oxidation and exchange reactions. In reducing environments, these disulfide bonds rapidly dissociated, resulting in redox‐triggered hydrogel degradation. This study demonstrated that the redox responsiveness of keratin hydrogels could be achieved by leveraging the inherent sulfhydryl groups of keratin.

Thirdly, disulfide bonds can be introduced either via intrinsic sulfhydryl groups in materials or through functionalization of raw materials, broadening the scope of hydrogel synthesis. Boz et al. [42] engineered a redox‐responsive hydrogel for tunable, “on‐demand” release of biomacromolecules. The terminal ends of PEG chains were modified with activated disulfide bonds and combined with four‐arm thiol‐functionalized PEG. The incorporation of sulfhydryl groups and disulfide bonds enabled redox responsiveness via sulfhydryl–disulfide bond exchange reactions. In environments containing high concentrations of thiol reagents (e.g., intracellular glutathione [GSH] or exogenous dithiothreitol), thiols attacked disulfide bonds, initiating exchange reactions that disrupted crosslinking, degraded the hydrogel, and released encapsulated drugs. Finally, disulfide‐bond hydrogels are advantageous for their simplicity and ease of functional modification, making them widely applicable in hydrogel design. Xu et al. [43] synthesized HA–SS–HA hydrogels via a simple mixing process. Rwandamuriye et al. [44] developed disulfide‐crosslinked hydrogels that exhibited accelerated degradation under reducing conditions. The disulfide‐crosslinked hydrogel's core advantage lies in its redox‐responsive nature [45]. The reductive substances within tumor cells provide an ideal trigger for intracellular drug release [46]. The dynamic reversibility of these bonds also confers self‐healing properties, facilitating injectable administration. However, in a highly recovered tumor microenvironment (TME), its structure may become unstable, leading to premature drug release. Consequently, it excels in combination therapies that require “on‐demand” and rapid drug release, but may be inferior to other strategies for tumor vaccine platforms that depend on sustained and stable signaling.

#### 2.1.4 C = N Bond

The formation of a C = N bond through the reaction between a carbonyl group and a nitrogen nucleophile is commonly referred to as a “click‐chemistry” reaction [47, 48]. This class of reactions exhibits several advantages: (1) it is operationally simple; (2) it constitutes a biologically orthogonal process with high chemical specificity, causing no interference with endogenous biological systems; and (3) water is the sole by‐product [49]. C = N bonds are formed via condensation reactions between carbonyl compounds (aldehydes or ketones) and diverse nitrogen nucleophiles, and their classification depends on the type of amine involved: (1) the condensation of a primary amine (R‐NH_2_) with a carbonyl group yields an imine (Schiff base, R_2_C = N‐R′); (2) the condensation of hydrazine (NH_2_‐NH_2_) with a carbonyl group produces a hydrazone (R_2_C = N‐NH_2_); and (3) the condensation of hydroxylamine (NH_2_‐OH) with a carbonyl group forms an oxime (R_2_C = N‐OH). The acid stability of these bonds varies significantly, conferring distinct functional properties to hydrogels [50, 51].

Hydrogels crosslinked by imine bonds exhibit instability in weakly acidic environments. Gu et al. [52] engineered a pH‐responsive hydrogel by conjugating the amino groups of silk fibroin and CS with dibenzaldehyde‐functionalized PEG via pH‐labile imine bonds. The hydrogel was nearly completely degraded within 24 h in a pH 5.0 solution but remained stable for 48 h in a pH 7.4 environment. This pH‐dependent degradation profile enables targeted drug release in the acidic TME. In this study, the cleavage of imine bonds under acidic conditions facilitated pH‐responsive drug delivery from the hydrogel. Conversely, hydrogels crosslinked by hydrazone bonds demonstrate stability in weakly acidic environments. Chang et al. [53] developed a hydrazone‐linked hydrogel by crosslinking acylhydrazide‐functionalized carboxymethyl cellulose with oxidized pectin through hydrazone formation between hydrazide and aldehyde groups. When hydrochloric acid was incrementally added to the hydrazone‐linked hydrogel, it maintained its gel state until the pH dropped below 3. Upon adjusting the system pH to 6–7, the solution re‐gelled, demonstrating the hydrogel's stability in the acidic TME.

The synthesis of C = N bonds is operationally straightforward and highly tunable. By modulating the feed ratio, the gelation time, pore size, degradation rate, and mechanical strength of C = N‐bond‐based hydrogels can be precisely controlled. Sun et al. [54] reported a hydrogel synthesized from N‐succinyl CS and oxidized dextran (ODEX) via carbonyl click chemistry. The gelation time decreased markedly from 3 min to 14 s as the precursor solution concentration increased, enabling the selection of an optimal gelation time that balances injectability and localized retention to prevent off‐target drug migration. This study demonstrated that feed concentration governs the gelation kinetics of C = N‐bond hydrogels. Additionally, Liu et al. [55] verified that the mass ratio of precursors influences the pore degradation time and mechanical properties of such hydrogels. A dynamic covalent hydrogel was fabricated by crosslinking 4‐arm‐PEG‐ONH_2_ with ODEX via C = N bonds between aldehyde and aminooxy groups. Hydrogels with higher precursor mass ratios exhibited smaller pore sizes, prolonged degradation times, and enhanced mechanical strength, whereas lower ratios produced the inverse effects. The tunable physicochemical properties of these hydrogels allow for tailored designs to meet specific application requirements. The reaction conditions for C = N bond formation are mild and chemically specific, enabling execution under physiological conditions. This characteristic makes C = N‐bond‐based hydrogels highly suitable for constructing stimuli‐responsive systems. Guedes et al. [56] developed an acid‐base‐sensitive hydrogel via imine linkage, while He et al. [57] demonstrated that gelation time in Schiff base‐mediated hydrogels can be regulated by adjusting the concentration of precursors. The pH‐sensitive nature of the C = N bond makes them a highly attractive strategy for targeted drug delivery within the TME. Their degradation products are typically biocompatible. Different types of C = N bonds offer varying degrees of acid stability, allowing for customization according to the specific pH of a target tumor. For instance, imine‐bonded hydrogels are well suited for the rapid release of checkpoint blockade antibodies in the acidic TME, thereby promptly reversing immunosuppression. However, their relatively low mechanical strength compromises their use in mechanically stressed environments. They serve as ideal carriers for both tumor vaccines and checkpoint blockade therapies, as both strategies rely on an intelligent response to the TME.

#### 2.1.5 Ester Bond

Ester bonds are typically formed via dehydration condensation reactions between carboxylic acids and alcohols [58]. The high polarity of the carbonyl carbon atom and the oxygen atom in the alcohol group renders ester bonds susceptible to hydrolytic cleavage [59]. In hydrogel synthesis, materials containing carboxyl and hydroxyl groups are widely employed, enabling the formation of biocompatible, thermodynamically stable ester crosslinks and broadening the applicability of ester‐bond hydrogels across diverse biomedical contexts [60, 61].

First, as a key characteristic of the TME, reactive oxygen species (ROS) play a crucial role in tumor initiation, progression, and therapy resistance [62, 63]. Abnormal metabolism and signaling in tumor cells lead to significantly elevated ROS levels, a feature that provides unique opportunities for designing tumor‐targeted drug delivery systems [64]. Against this background, ester bond‐based hydrogels stand out due to their ROS‐responsive degradation mechanism. As functional groups sensitive to oxidative environments, ester bonds undergo selective cleavage under high ROS concentrations, thereby enabling specific drug release at the tumor site. This characteristic not only significantly enhances the targeting efficiency of therapeutic agents but also improves antitumor efficacy by modulating drug release kinetics, while simultaneously reducing systemic toxicity to normal tissues. Specifically, ester bonds are susceptible to attack by ROS. Consequently, under ROS‐rich conditions, the progressive cleavage of ester bonds allows hydrogels crosslinked via ester linkages to degrade and release encapsulated drugs in a ROS‐responsive manner. Sun et al. [65] developed an injectable, ROS‐degradable therapeutic hydrogel in which ester bonds exhibit ROS reactivity, enabling their gradual cleavage under oxidative stress to achieve sustained drug release. Hydrogel incubation with hydrogen peroxide (H_2_O_2_) at varying time points demonstrated significant drug release after 10 days, confirming its ROS‐responsive degradation profile. Subsequent studies have corroborated this mechanism. Zhang et al. [66] fabricated a polyacrylic acid‐based hydrogel by crosslinking pullulan with poly(deca‐4,6‐diynedioic acid) (PDDA). Carboxyl groups on PDDA reacted with hydroxyl groups on pullulan to form ester bonds. The hydrogel's degradation rate, responsive to in vivo ROS levels, enables controlled and prolonged drug delivery.

The formation of ester bonds is rapid and operationally straightforward. This process requires simply mixing the raw materials, injecting them into the target site, and allowing a few minutes for gelation. An in situ hydrogel can then form to deliver therapeutic agents effectively. Dai et al. [67] developed a biocompatible, degradable hydrogel scaffold with tunable properties for targeted drug delivery. By sequentially injecting a drug‐loaded TSPBA solution followed by a PVA solution into the tumor site, an in situ hydrogel was rapidly generated. The localized distribution of the hydrogel enhanced the retention time of the encapsulated payloads. In this study, the sequential injection of precursor solutions confirmed the rapid gelation kinetics of the system. Additionally, research has explored the construction of glucose‐responsive in situ hydrogels by leveraging the reactivity of ester bonds to fluctuating glucose concentrations. Tong et al. [68] synthesized an in situ hydrogel by grafting phenylboric acid‐functionalized γ‐PGA (PBA–PGA) onto diol groups of konjac glucomannan to form dynamic ester bonds. These bonds were engineered to respond to in vivo glucose levels: under acidic conditions, ester bonds coordinated with vicinal diol groups of glucose molecules. During hyperglycemic states, glucose diffusion into the hydrogel matrix triggered ester bond cleavage, enabling rapid drug release. In gel material synthesis, ester bond formation provides rapid reaction kinetics and favorable mechanical properties, making it an ideal strategy for applications requiring immediate crosslinking or prototyping. Kim et al. [69] fabricated in situ hydrogels by coinjecting polyphenylboric acid and mannan into tumors using dual syringes. Zhou et al. [70] similarly developed ester bond‐based hydrogels capable of conforming to irregular surgical incisions through in situ gelation. The key feature of ester‐bond‐crosslinked hydrogels lies in their responsiveness to ROS. This property enables ROS‐triggered, targeted drug release within the TME, facilitating synergistic effects in combination therapies. Additionally, the formation of ester bonds is typically rapid and straightforward. However, the inherent instability of these bonds also constitutes a major weakness, as premature degradation can occur, leading to unintended burst release and a shortened depot effect. Therefore, while these hydrogels are highly effective in systems designed for short‐term release to rapidly alter the TME, they are unsuitable as carriers for therapies requiring steady, long‐term release.

#### 2.1.6 Amide Bond

Amide bonds are formed via condensation reactions between carbonyl compounds and amino groups, a ubiquitous process in natural systems [71]. In hydrogel fabrication, amide bonds exhibit excellent biocompatibility and moderate acid stability, which prolong the functional lifespan of the hydrogel. Additionally, amide bonds enhance the hydrophilicity and surface charge distribution of materials, promoting cell adhesion—a critical feature for tumor immunotherapy applications.

The stability of amide bonds imparts structural robustness to hydrogels. Specifically, amide bonds confer resistance to enzymatic degradation. For instance, Pornchanok et al. [72] utilized amide bonds to crosslink's amino groups with succinic acid's carboxyl groups. When immersed in PBS containing 1.5 µg/mL lysozyme for 6 weeks, the uncrosslinked CS exhibited significantly greater weight loss compared with the amide‐crosslinked hydrogel. In this study, the enzymatic resistance of amide bonds resulted in a slower drug release profile. Furthermore, amide bonds enhance acid stability, preserving hydrogel integrity and extending therapeutic efficacy in acidic environments. Zheng et al. [73] reported a carboxymethyl CS (CMCh) hydrogel crosslinked via amide bonds between amino and carboxyl groups under EDC/NHS catalysis. This hydrogel demonstrated slower degradation in pH 5.4 solutions than in pH 7.4 solutions, indicating its prolonged retention in acidic TMEs for sustained drug delivery and tumor therapy. In vivo, moderate acid stability prevents premature hydrogel degradation, extending the effective lifespan of implants or carriers and enhancing therapeutic outcomes. Stable amide bonds also maintain structural integrity, which is essential for supporting cell growth and adhesion. Bu et al. [74] fabricated adhesive hydrogels by crosslinking tetra‐PEG‐NH_2_ with tetra‐armed PEG succinimidyl succinate via amide bonds. Upon contact with tissue, additional amide bonds formed between the hydrogel and tissue surfaces, achieving adhesive strengths (∼20 kPa) surpassing those of commercial fibrin glue. This strategy enabled strong tissue‐hydrogel adhesion through covalent bonding. Recent advancements further optimize adhesion, Strehin et al. [75] designed an 8‐arm PEG succinimidyl glutarate and Cys‐terminated 8‐arm PEG network crosslinked via amide bonds between carboxyl and amino groups. This adhesive hydrogel achieved a shear strength of 46 ± 8 kPa after 1 h of gelation, effectively bonding tissue surfaces. The acid stability of amide bonds, combined with their adhesive properties, offers significant advantages for biomedical applications. Yu et al. [76] demonstrated that amide‐crosslinked hydrogels retained over 50% of their mass after 21 days in PBS, while Wang et al. [77] reported stable crosslinking structures formed by direct precursor mixing. These studies highlight the versatility of amide bonds in creating durable, functional hydrogels for biomedical use. The amide bond is one of the most stable and prevalent chemical linkages in nature. This inherent stability confers hydrogels with exceptional mechanical strength, resistance to enzymatic degradation, and long‐term stability under physiological pH [78]. These properties make them an ideal choice for applications that require long‐term implantation and maintenance of structural integrity. Their excellent cell adhesiveness further facilitates the repair and integration of local tissues. However, this high stability also renders the hydrogels biologically nonresponsive, making them difficult to degrade rapidly in response to specific biological signals. This may lead to incomplete drug release or even necessitate a secondary surgical procedure for removal. Therefore, while amide‐bond‐crosslinked hydrogels excel in applications such as long‐term immune modulation. For instance, they perform exceptionally well as sustained‐release systems for vaccine adjuvants and are also well suited as tissue engineering scaffolds to support immune cell colonization. But their utility is limited in dynamic therapies that require intelligent feedback and adaptive drug release.

#### 2.1.7 Coordinate Bond

Coordination bonds are a distinct type of covalent bond in which the shared electron pair originates from a single atom [79]. In conventional hydrogels, the electron cloud of coordination bonds tends to localize toward atoms with higher electronegativity, resulting in pronounced polarity [80]. Consequently, the stability of coordination bonds is largely determined by the interaction between the ligand and the central metal ion [81]. These bonds impart unique properties to hydrogels, enabling diverse applications in biomedical and functional material design.

Some coordination bonds exhibit high sensitivity to temperature fluctuations. As temperature increases, intensified molecular motion can disrupt weaker coordination bonds, transitioning the hydrogel from a gel state to a sol phase. Conversely, cooling promotes the reformation of these bonds, facilitating sol‐to‐gel transition. This thermoresponsive sol–gel phase shift endows hydrogels with temperature‐dependent functionality. Cao et al. [82] synthesized a CMCh‐based hydrogel by introducing a minimal amount of Fe^3^^+^ into an aqueous CMCh solution. The carboxyl groups in CMCh formed coordination bonds with Fe^3^^+^, generating a crosslinked CMCh–Fe hydrogel network. The dynamic and reversible nature of these bonds enabled the hydrogel to exhibit thermoresponsive behavior, undergoing reversible gel–sol transformations with temperature changes. Furthermore, the reversibility of coordination bonds allows damaged hydrogel networks to self‐repair by reestablishing broken bonds without external intervention, conferring excellent self‐healing capabilities. Yang et al. [83] exploited the dynamic reversibility of coordination bonds to fabricate a self‐healing hydrogel by crosslinking carboxymethyl cellulose–ethylenediamine–gallic acid (CEG) with Fe^3^^+^ via interactions between Fe^3^^+^ and CEG hydroxyl groups. By adjusting the pH, the number of coordination bonds was modulated, directly influencing the hydrogel's self‐healing efficiency and mechanical strength. Coordination bonds can chemically anchor drugs to the hydrogel matrix, enabling effective drug loading. Meanwhile, the reversible dissociation of these bonds allows for sustained and controlled drug release, maintaining an effective drug concentration at the tumor site. Shen et al. [84] utilized coordination bonds to link the drug Elesclomol (ES) with copper ions (Cu^2^^+^) and the alginate chains. This approach anchored the hydrophobic ES–Cu complex onto the hydrophilic alginate backbone, forming an injectable pre‐gel solution. This method enabled the active immobilization and controlled release of the drug, while the coordination interaction also allowed the hydrophobic ES–Cu to remain stable within the hydrogel system, thereby increasing both the drug loading capacity and the stability of the formulation. In other study, Wu et al. [85] constructed a carrier‐free ternary hydrogel through self‐assembly mediated by metal‐coordination bonds and hydrogen bonds using glycyrrhizic acid, Cu^2^^+^, and celastrol (Cel). This strategy successfully incorporated the lipophilic Cel into a stable hydrogel for the first time, effectively addressing its poor water solubility and high toxicity. The resulting hydrogel modulates the TME via chemodynamic therapy and cuproptosis, promotes T‐cell activation and infiltration, and synergistically suppresses both primary and metastatic tumors when combined with aPD‐L1 treatment.

In summary, the dynamic and reversible characteristics of coordination bonds not only enable sol–gel phase transitions but also confer self‐healing and thermoresponsive properties to hydrogels, significantly broadening their applicability and technical versatility. Coordinated‐bond‐based hydrogels exhibit versatile functionality, including thermoresponsiveness, self‐healing capability, and the capacity to serve as effective payload platforms for therapeutic metal ions and hydrophobic drugs [86, 87]. These characteristics render them highly promising for combination therapies. However, the potential biosafety concerns associated with metal ions represent a major challenge for clinical translation, necessitating careful selection of metal species and their concentrations. These hydrogels hold considerable promise for constructing sophisticated, multimodal immunotherapy platforms, particularly for integrating immunotherapy with metal ion‐mediated treatment strategies.

### 2.2 Physical Crosslinking

Chemical crosslinking can rapidly form hydrogels under mild conditions, but it usually requires the addition of ionic crosslinking agents with certain toxicity to promote the reaction process, thereby limiting the application of chemical crosslinked hydrogels. Physical crosslinked hydrogels are mainly formed through electrostatic interactions, hydrogen bonds, hydrophobic interactions and van der Waals forces between polymers, without the addition of ionic crosslinking agents, and thus have broad application prospects [88].

#### 2.2.1 Electrostatic Interaction

Electrostatic interaction is a fundamental mechanism in forming physically crosslinked hydrogels, arises from the attractive or repulsive forces between charges. This category includes ionic bonds, dipole–dipole interactions, and ion–dipole interactions [89]. As reversible, dynamic, and noncovalent bonds, electrostatic interactions are particularly valuable for constructing smart hydrogels characterized by self‐healing, injectability, and responsive properties [90].

Conventional chemical crosslinking methods often introduce cytotoxic components, restricting the use of hydrogels as carriers for drugs or nutrients [91]. Electrostatic interaction offers a promising alternative to overcome this limitation. As a physically crosslinking strategy, it enables the fabrication of intelligent, responsive hydrogels without compromising their inherent cytocompatibility and biodegradability [92]. Ma et al. [93] constructed fucoidan (FC)–GS magnetic aerogel microspheres by forming a polyelectrolyte complex through electrostatic interactions between FC and CS. These microspheres selectively loaded cationic drugs via electrostatic adsorption and achieve controlled release through pH‐responsive behavior and magnetic targeting. The system offered several advantages, including selective drug loading, magnetic targeting and recovery, providing a versatile platform for targeted drug delivery in cancer immunotherapy. Utilizing pH‐dependent electrostatic interactions, the gel microspheres can selectively adsorb and load positively charged cationic drugs, enabling selective loading and pH‐triggered release of drugs, which can be applied for the delivery of immunomodulators. Falcone et al. [94] utilized a smart release hydrogel via the electrostatic interaction between the laponite and gelatin to load the immune‐checkpoint inhibitor aPD‐1 and doxorubicin (DOX) for the treatment of hepatocellular carcinoma (HCC). DOX release sharply when the hydrogel reached mild acid tumor environment because laponite and DOX have the same charge at pH 5–6. While aPD‐1 release slower along with the degradation and swelling of hydrogel. This smart hydrogel provided an ideal function mode for chemotherapy, which could release chemotherapy drugs first to convert the tumor to a “hot” tumor, followed by the release of immunotherapeutic agents to establish a second line of therapeutic defense.

The formation of classic alginate‐based hydrogels is fundamentally based on ionic bonds, which create a three‐dimensional network through electrostatic interactions between negatively charged alginate polymer chains and positively charged multivalent cations [95]. Alginate solution could be injected directly into tumor sites, where endogenous Ca^2^^+^ ions facilitated in situ hydrogel formation through ionic bonds. Xiao et al. [96] developed an injectable, in situ‐forming hydrogel by mixing sodium alginate with nanoparticles coloaded with glucose oxidase and R848, which is ionically crosslinked by physiological Ca^2^^+^. This formulation enables localized retention and controlled release of therapeutics at the tumor site. The hydrogel orchestrates a combination immunotherapy by synergizing starvation therapy and repolarization of tumor‐associated macrophages to the antitumor M1‐like phenotype. Sun et al. [97] similarly created an in situ hydrogel by crosslinking sodium alginate with endogenous Ca^2^^+^, which formed ionic bonds between Ca^2^^+^ and alginate carboxyl groups. The resulting hydrogel remained localized within the tumor and enabled repeated low‐dose drug release over an extended period. Similarly, in another study, Li et al. [98] developed a 3D‐printed hydrogel scaffold using electrostatic interactions between anionic sodium alginate and Ca^2^^+^ to load the STING agonist MSA‐2. This strategy provided excellent printability and structural stability for controlled, sequential drug release. In the above examples, unlike other weak physical interactions, ionic bonds create a network that is both stable and dynamic. The bonds can break and re‐form under certain conditions, giving the gel self‐healing and shear‐thinning properties. Electrostatically crosslinked hydrogels form under exceptionally mild conditions, eliminating the need for chemical crosslinkers and thereby exhibiting outstanding biocompatibility [99]. This makes them particularly suitable for encapsulating sensitive biomacromolecules and even living cells. Their inherent self‐healing and shear‐thinning properties ensure excellent injectability and effective filling of irregular tumor cavities [100]. However, the main limitation is their relatively low mechanical strength, as they may gradually decompose in a physiological environment due to ion exchange [101]. These hydrogels are widely used for the local delivery of various immunotherapeutic agents, especially in scenarios that require the rapid in situ formation of a “drug depot” without compromising drug activity.

#### 2.2.2 Hydrogen Bond

A hydrogen bond is a special intermolecular or intramolecular interaction formed between X and Y when a hydrogen atom approaches an atom Y with a large electronegativity and a small radius, mediated by hydrogen. Hydrogen bonding stands as a highly advantageous strategy for constructing hydrogels, primarily due to its dynamic and reversible nature. These noncovalent bonds can spontaneously reform after rupture, endowing the hydrogels with remarkable self‐healing properties and outstanding injectability through shear‐thinning behavior. Furthermore, the responsiveness of hydrogen bonds to stimuli enables precise control over gelation.

Common DNA hydrogels are formed by crosslinking DNA through hydrogen bonds. When designing the DNA sequence, sticky ends are left to allow crosslinking through base complementary pairing [102]. Zhang et al. [103] utilized DNA as precursor to construct the 3D network via the crosslinking of complementary base pairing, which is based on hydrogen bond. The programmability of DNA sequence facilitates the tunable crosslinking site of hydrogel and thus form tunable pore size for different application. The SEM results shows that the DNA hydrogel presented 3D and porous network structure with mean pore size of around 13.67 µm, which was larger than the size of T cells and was beneficial to cell diffusion and enrichment. In the above examples, hydrogen bonds provide sufficient structural stability for the hydrogel, protecting the stability of the drug encapsulated in the hydrogel. Meanwhile, the reversibility of hydrogen bonds enables the hydrogel to respond to specific environmental signals and achieve targeted drug release. Wang et al. [104] engineered a DNA hydrogel via the hydrogen bond between complementary bases. Due to the high presence density of nitrogen atoms, oxygen atoms, and hydrogen atoms, the hydrogen bond could be formed between different nucleus such as DNA and siRNA, which facilitate the carry of gene therapy. Additional, due to the ubiquitous hydrogen bond, even when the DNA chain break, it could still form into a self‐assembly nanoparticle, which could facilitate the delivery and protection of gene agents. In another study, Zhao et al. [105] also constructed an aptamer‐based hydrogel by leveraging interstrand base‐pairing hydrogen bonds in DNA. This hydrogel enabled the sustained release of catalysts for over four days, effectively facilitating the generation of hydroxyl radicals to induce immunogenic cell death (ICD). This system provided a robust platform for tumor chemodynamic–immunotherapy.

Hydrogen bonding is an important mechanism for constructing peptide‐based hydrogels and is the fundamental for realizing the functions of such hydrogels. The hydrogen bonding effect has enabled researchers to successfully construct thermally responsive injectable peptide hydrogels suitable for biomedical applications. Li et al. [106] constructed a thermosensitive hydrogel using methoxy poly(ethylene glycol)‐block‐poly(l‐alanine) (mPEG‐b‐PAla) polypeptide material, driven by hydrogen bonding and hydrophobic interactions within β‐sheet structures. This design makes the hydrogel injectable and thermosensitive, allowing it to rapidly form a gel at body temperature for local drug delivery. In tumor immunity, this hydrogel acts as a drug carrier to achieve the sequential release of regorafenib and the TGF‐β inhibitor LY3200882, effectively inhibiting tumor growth and metastasis. Song et al. [107] grafted l‐valine onto PEG to create a thermoresponsive complex capable of forming an in situ hydrogel in vivo. To evaluate its gelation capacity, the sol–gel phase transition temperature was systematically investigated. Results indicated that the higher the concentration of the precursor solution, the faster the gelation after subcutaneous injection.

Beyond its established role as a primary construction strategy in DNA and peptide hydrogels, hydrogen bonding is also extensively utilized in various other types of hydrogel systems, particularly those based on synthetic and natural polymers. In many hydrogels formed from synthetic polymers, hydrogen bonding serves as the core driving force for gelation. In the study by Jangizehi et al. [108], hydrogels were formed by polymerizing N‐acryloyl glycinamide (NAGA) monomers in aqueous solution, with the network structure relying on intermolecular hydrogen bonds between NAGA units. The application of hydrogen bonding enables the construction of robust supramolecular hydrogels, where hydrogen bonds remain stable in water through the formation of microdomains, endowing the hydrogels with capabilities such as reversible swelling/deswelling, self‐healing, and reshapeability. In another study, Wu et al. [109] prepared hydrogen‐bonded PVA–urea hydrogels by drying PVA–urea solutions and subsequently swelling them at elevated temperatures. The reversible formation and breakage of hydrogen bonds provide the hydrogels with an efficient energy dissipation mechanism, resulting in excellent mechanical properties. The obtained hydrogels, characterized by high strength and modulus, are suitable for various applications including soft tissue engineering. In summary, hydrogen bonding represents a crucial strategy in hydrogel design, particularly for fabricating supramolecular hydrogels with dynamic properties such as self‐healing, injectability, and reshapeability. Hydrogen‐bond‐crosslinked hydrogels, particularly those based on DNA and polypeptides, offer unparalleled advantages in bio‐mimicry and biospecificity [110]. The programmable nature of DNA hydrogels allows for precise control over pore size, degradation kinetics, and drug release sequences, which is crucial for orchestrating complex immune cell interactions [111, 112]. Conversely, polypeptide hydrogels can mimic the natural extracellular matrix, providing an ideal 3D microenvironment for encapsulating and delivering adoptive T cells. However, the relatively weak nature of hydrogen bonds often results in poor mechanical properties and can pose stability challenges within complex bodily fluids. Despite this, the strategy is a top choice for cell delivery, personalized cancer vaccines, and advanced therapies demanding a high degree of biological recognition.

#### 2.2.3 Hydrophobic Interaction

Hydrophobic interactions refer to the attractive interactions between the hydrophobic parts of a system [113]. Hydrophobic interaction is a critical mechanism for constructing high‐performance hydrogels. It enhances the network through the formation of hydrophobic microdomains, which simultaneously suppress excessive swelling and improve mechanical strength and responsive behavior [114]. In practice, hydrophobic interaction often synergizes with other noncovalent bonds to achieve multifunctional, high‐performance hydrogels, making it an indispensable strategy for applications demanding high strength, antiswelling capability, and intelligent responsiveness [115, 116].

Pluronic F127 is an amphiphilic triblock copolymer that exhibits a temperature‐dependent sol–gel transition. At elevated temperatures, the hydrophobic poly(propylene oxide) (PPO) segments aggregate via hydrophobic interactions, forming the micellar cores, while the hydrophilic poly(ethylene oxide) (PEO) segments constitute the hydrated coronas [117, 118]. These micelles then pack into an ordered three‐dimensional network, resulting in macroscopic gelation. Kim et al. [119] utilized Pluronic F127‐grafted gelatin to form hydrogel via hydrophobic interaction at body temperature. When the temperature raised, the thermal motion of water molecules intensified, and the hydrophobic PPO segments began to aggregate relying on hydrophobic interactions in order to minimize the contact area with water. This thermal controlled force change endows hydrogel thermal response and good injectablity, which could be sol state at low temperature while be gel state at body temperature. The exclusive reliance on hydrophobic interactions for physical crosslinking in the aforementioned examples allows for gelation at extremely low concentrations and the simultaneous, spontaneous formation of uniformly sized drug‐loaded micelles. This ultimately confers the unique and critically important lymphatic targeting capability. Fan et al. [120] developed an injectable and adhesive hydrogel using thermosensitive PND nanogels containing catechol groups, which assemble through hydrophobic interactions. This design allows the hydrogel to form in situ at body temperature, acting as an “antigen reservoir” that captures tumor antigens released by PTT. It thereby continuously recruits and activates dendritic cells (DCs), enhancing CD8^+^ T cell‐mediated antitumor immunity and effectively inhibiting the growth of primary, distant, and rechallenged tumors. The nanogels form hydrogel via hydrophobic interactions at body temperature, providing thermal sensitivity and injectability for in situ antigen capture. In some other studies, hydrophobic interactions have also been widely applied in the construction of hydrogels, endowing them with temperature sensitivity and injectability. Li et al. [121] developed a bioadhesive hydrogel by conjugating dopamine to Pluronic F127 via Michael addition. The modified hydrogel exhibited strong tissue adhesion, making it suitable for postsurgical wound closure. Meng et al. [122] exploited the thermoresponsive nature of Pluronic F127 as a drug depot for codelivering multiple therapeutic agents. Hydrogels exhibited strong thermal reversibility.

Hydrophobic interactions serve as the cornerstone for constructing functional injectable hydrogel platforms. They endow materials with thermosensitivity and injectability, while also acting as physical crosslinking points that constitute the gel network [123]. Their reversible and tunable nature is further exploited as a key feature for achieving intelligent, controlled drug release. In the study by Jia et al. [124], a PDLLA–PEG–PDLLA hydrogel was designed in which the thermosensitive sol–gel transition is entirely driven by hydrophobic interactions. Leveraging the temperature sensitivity of these interactions enabled remote, spatiotemporally controlled drug release. Furthermore, the hydrophobic microdomains function as reservoirs for hydrophobic drugs such as R848, facilitating sustained release. In another study, Shi et al. [125] developed a thermosensitive hydrogel based on poly(ethylene glycol)‐block‐poly(γ‐ethyl‐l‐glutamate) (mPEG‐b‐PELG) as a drug delivery system for the sustained release of chemotherapeutic agents and immune checkpoint inhibitors. Here, the temperature responsiveness of hydrophobic interactions was engineered as a “switch” to regulate drug release, enabling smart, on‐demand drug delivery. Hydrophobic interaction is the key mechanism for achieving thermosensitive physical hydrogels. These hydrogels belong to intelligent responsive hydrogels, and their gelation behavior is regulated by temperature. They are suitable for local drug delivery systems. Hydrophobic interaction endows hydrogels with important functions such as injectable, thermosensitive gelation, and near‐infrared light‐triggered release, which is the basis for achieving local photothermal immunotherapy. Hydrophobic interactions serve as the primary driving force for the formation of thermogelling hydrogels. Their characteristic inverse thermogelation behavior enables minimally invasive injection and in situ formation of antigen or drug depots. The hydrophobic microdomains within these gels provide an ideal environment for hosting hydrophobic immunomodulators. However, a major limitation lies in their generally weak mechanical strength and the risk of dissolution in vivo due to dilution below the critical micelle concentration, which can significantly shorten the depot's functional duration. Consequently, this system is highly effective for rapidly establishing localized, thermally triggered drug release.

#### 2.2.4 Crystallization Domain Crosslinking

Crystalline domain crosslinking is a hydrogel design strategy that relies on the formation of uniformly distributed crystalline domains within the hydrogel network, serving as dynamic crosslinking sites. This approach enables an effective balance between stiffness and toughness, allowing the hydrogel to exhibit both high rigidity and high fracture resistance, thereby broadening its potential for biomedical applications.

PVA hydrogel is a typical example of a system structured through crystalline domain crosslinking. It utilizes crystalline regions formed between PVA molecular chains as physical crosslinking points to establish a three‐dimensional network and achieve gelation. Jiang et al. [126] employed a variable‐temperature solvent exchange strategy to regulate this process. In the first stage, low temperature promoted hydrogen bond reconstruction between PVA chains, facilitating crystal nucleation. In the second stage, raising the temperature to room temperature enabled chain folding and arrangement on existing nuclei, promoting crystal growth. This resulted in a robust and resilient network crosslinked by well‐developed crystalline domains. Through precise control of crystallization nucleation and growth, gel materials with high modulus, strength, and toughness were obtained.

By carefully controlling the nucleation and growth stages of crystallization, a more optimized network architecture can be constructed, simultaneously enhancing modulus, strength, and toughness. Ren et al. [127] designed a dual‐network hydrogel composed of PVA, polysulfobetaine (PSBMA), and H_2_SO_4_. Leveraging the strong salting‐out effect (Hofmeister effect) of H_2_SO_4_ and the hydration capacity of PSBMA, they synergistically induced PVA to form numerous nanocrystalline domains as primary crosslinks. The obtained structure includes the interpenetration of a rigid PVA crystal network and a flexible PSBMA entanglement network, simultaneously achieving high strength and high toughness. In another study, Jiang et al. [128] constructed a crystalline domain‐crosslinked network via cyclic freeze‐thaw treatment of PVA, providing a flexible yet stable framework that maintained the structural integrity of the hydrogel. The reversible nature of crystalline crosslinking imparted excellent resilience and stability. Additionally, by incorporating an ionic coordination network based on CS alongside the PVA crystalline network, the hydrogel attained ultra‐high strength and toughness. The high mechanical strength offered by the crystalline crosslinked network provides a stable microenvironment for T‐cell survival, while the tunable density of crystalline domains allows modulation of drug diffusion rates. This enables sustained codelivery of antigens and adjuvants, eliciting a robust and durable systemic immune response. In summary, crystalline domain‐crosslinked hydrogels combine high strength, reversible self‐healing, good biocompatibility, and tunable properties, making them ideal local delivery platforms for tumor immunotherapy. Hydrogels crosslinked by crystalline domains are distinguished by their exceptional mechanical properties, achieving a unique combination of high strength and toughness. This provides a highly stable 3D microenvironment for supporting immune cell survival and function. Their slow and controllable degradation kinetics are well suited for the sustained codelivery of antigens and adjuvants to maintain long‐term immune responses. However, the fabrication process is often complex, and their degradation lacks responsiveness to biological signals. This strategy is best suited for applications that require a rigid scaffold, such as implantable devices for long‐term immunomodulation or high‐strength 3D cell culture models.

#### 2.2.5 Subject–Object Interaction

Host–guest complexation refers to a process driven primarily by hydrophobic forces, whereby a guest molecule is enclosed within the hydrophobic cavity of a host molecule. Upon hydrophobic aggregation of polymer chains into crystalline complexes, sol–gel transitions occur, leading to the formation of host–guest gels [129]. This specific interaction serves as a foundational tool for developing advanced smart materials. It enables the unique combination of high strength, fast self‐recovery, injectability, and responsiveness to diverse stimuli within a single hydrogel platform [130]. Consequently, these materials hold significant potential for controlled drug delivery, tissue engineering, and cell encapsulation, offering particular promise for precision applications such as dynamic cancer immunotherapy [131].

The α‐cyclodextrin/PEG (α‐CD/PEG) host–guest combination represents the most widely adopted system in tumor immunotherapy hydrogels. Its core advantage lies in its combination of PEG's inherent biocompatibility with α‐CD's dynamic reversible crosslinking capability, which collectively enable the hydrogel to achieve three critical functional properties: injectability, self‐healing characteristics, and sustained release profiles [132]. Yang et al. [133] demonstrated this phenomenon by preparing a hydrogel from PEG (PEG4000) and α‐CD. Under shear stress, the hydrogel transitions to a solution‐like state, reverting to its original structure upon stress removal. This shear‐dependent viscosity reduction confirms its injectability. Furthermore, the simplicity of physical crosslinking via host–guest interactions enables rapid gel formation through simple mixing. The α‐CD/PEG host–guest system represents the most widely adopted platform in tumor immunotherapy hydrogels. Its core advantage combines PEG's excellent biocompatibility with α‐CD's reversible supramolecular crosslinking, enabling injectability, self‐healing properties, and sustained antigen release. As demonstrated in the DC‐tumor cell vaccine platform, this hydrogel effectively promotes DC maturation and T‐cell activation through prolonged antigen presentation. Xu et al. [134] further optimized this system by incorporating mPEG–OVAp–AuNPs with α‐CD, where gold nanoparticles enhanced antigen presentation efficiency for amplified immune activation. The hydrogel's crosslinking mechanism relies on host–guest interactions between α‐CD cavities and PEG‐terminated hydrophobic groups, with dissociation kinetics precisely tunable via PEG chain length and α‐CD concentration. Compared with other systems, α‐CD/PEG hydrogels offer advantages including facile preparation, superior biocompatibility, and high drug‐loading capacity. This system provides a versatile platform for tumor immunotherapy, particularly suited for sustained antigen delivery strategies.

The β‐CD/ferrocene (β‐CD/Fc) host–guest system introduces a groundbreaking photoreponsive hydrogel platform for tumor immunotherapy. Xu et al. [135] developed a hyaluronic acid‐based β‐CD/Fc hydrogel that leverages the high‐affinity interaction between β‐CD and Fc to create a stable network, with light‐triggered dissociation enabling spatiotemporally controlled drug release. This system achieves precise immunomodulator delivery through light stimulation, effectively preventing tumor recurrence and metastasis after surgical resection. The β‐CD/Fc system's key innovation lies in its dual functionality: high binding stability ensures structural integrity, while the photoreponsive mechanism allows therapeutic release on demand.

The α‐CD/cationic polymer host–guest system has demonstrated unique advantages in tumor immunotherapy, particularly for applications requiring cell engineering and gene delivery. Zhu et al. [136] developed a CD3–PPP/pCAR@α‐CD hydrogel that utilizes the supramolecular interaction between α‐CD and CD3–PPP cationic polymers to create a sustained‐release “cell factory” capable of continuously producing pCAR plasmids. This system enables continuous in vivo generation of chimeric antigen receptor T cells (CAR‐T cells) without requiring external stimuli, relying instead on gradual hydrogel degradation to release its cargo in alignment with natural physiological processes. As an innovative strategy for tumor immunotherapy, this platform holds particular promise for long‐term cell therapy applications and represents a significant advancement in advancing in vivo cell treatment technologies. Host–guest interactions serve as a paradigm for constructing supramolecular hydrogels, seamlessly integrating exceptional dynamic properties with a high degree of functional designability [137]. Different host–guest pairs introduce responsiveness to diverse stimuli, while their mild assembly conditions are inherently gentle toward cells and biomolecules [138]. A primary limitation, however, is their generally moderate mechanical strength, coupled with the sometimes cumbersome synthesis of functionalized host or guest molecules. This approach excels in applications that demand highly dynamic and intelligent materials, such as sustained and responsive delivery of CAR‐T cells, and injectable tumor vaccine platforms with self‐healing capabilities.

### 2.3 Design Principles of Crosslinking Strategies for Hydrogels

In the design of immunotherapeutic hydrogels, the selection of crosslinking strategy is paramount as it directly determines the material's physicochemical properties and its interactions with the immune system. As two fundamental fabrication methodologies, chemical and physical crosslinking offer distinct technical advantages and application prospects for immune regulation. Chemical crosslinking creates stable three‐dimensional networks through covalent bonds, typically exhibiting superior mechanical strength and long‐term stability. In contrast, physical crosslinking relies on noncovalent interactions, resulting in more dynamic and reversible network architectures [139, 140].

For therapeutics requiring sustained release, such as in cancer vaccines, chemically crosslinked hydrogels are often the superior choice. Their covalently stabilized 3D network effectively protects fragile antigens from rapid degradation [141]. By modulating the hydrogel's degradation rate, sustained drug release over several weeks can be achieved. This prolonged immunostimulation is crucial for activating and expanding antigen‐specific T cells to elicit a robust adaptive immune response [48]. Chemically crosslinked hydrogels are also more suitable for scenarios demanding structural barriers or fillers, such as in postsurgical immunotherapy coupled with tissue repair [142]. Their robust network can act as a physical barrier to isolate residual tumor cells, while their slow degradation properties enable them to function as a long‐term drug depot. Continuously release therapeutic drugs to eradicate residual lesions and activate local immunity, thereby effectively preventing tumor recurrence [143]. For strategies requiring the formation of an in situ drug reservoir at the tumor site, such as with checkpoint inhibitors, physically crosslinked hydrogels demonstrate distinct advantages. Their gelation process proceeds under mild conditions without harsh chemical reactions, which better preserves the bioactivity of macromolecular drugs like antibodies [144, 145]. Furthermore, their excellent injectability facilitates the formation of a localized drug depot for controlled, regional release, significantly reducing systemic exposure to checkpoint blockers and their associated toxicities [146]. Physically crosslinked hydrogels are also more applicable for strategies providing temporary niches for cell infiltration, as in adoptive cell therapy [147]. Their ExtraCellular Matrix‐mimetic and gentle gelation environment supports high cell viability, while their inherent porous structure facilitates the migration and infiltration of adoptive T cells [148]. Meanwhile, chemical and physical crosslinking are not mutually exclusive choices but often synergistic and complementary. For instance, enzymatically catalyzed chemical crosslinking can rapidly construct a stable covalent network skeleton, while subsequently formed intermolecular forces such as hydrogen bonding and hydrophobic interactions further consolidate and optimize this network structure. They collectively enabling precise regulation of drug release kinetics [149, 150, 151]. As two core preparation methods, chemical and physical crosslinking provide unique technical advantages and application prospects for immunomodulation precisely through this multilevel synergistic interaction.

Therefore, the core design principle for immunotherapeutic hydrogels lies in the precise matching of the crosslinking strategy to the specific requirements of the application. As our understanding of immune system mechanisms deepens and material synthesis technologies advance, rationally designed hydrogel platforms are poised to significantly propel the field of cancer immunotherapy forward.

## 3 Application Strategy

In tumor immunotherapy, hydrogels have emerged as versatile delivery vehicles for diverse pharmaceutical agents. Immune checkpoint inhibitors, CAR‐T cells, antibodies, and adjuvants are routinely employed in clinical settings. Immune checkpoint inhibitors modulate T‐cell inhibitory signals to enhance antitumor immunity, while CAR‐T cells directly target tumor cells [152, 153]. Adjuvants activate DCs and facilitate antigen presentation [154]. Hydrogels are increasingly adopted as delivery systems in tumor immunotherapy. This review systematically classifies and elaborates on current hydrogel applications based on immune strategies. Additionally, the advantages of hydrogels in specific contexts were summarized, emphasizing their multifunctional roles as precision delivery platforms, immune microenvironment modulators, and cell therapy support systems. These application strategies are summarized in Table 2.

**TABLE 2:** Hydrogel application strategies.

Category	Application strategy	Advantages	Representative examples
Protein/peptide	Improving oral delivery and bioavailability	Protects proteins/peptides from enzymatic degradation, prolongs systemic retention, improves bioavailability	Ouyang et al., Journal of the American Chemical Society, 2023. Calcium alginate hydrogel encapsulated selenium nanoparticles to enhance the oral bioavailability of selenoproteins [175].
			Zhou et al., Advanced Materials, 2021. The zwitterionic hydrogel (MOF‐Gel) enhanced the oral bioavailability of exendin‐4 [180].
	Augmenting immune responses	Induces ICD, activates T cells, enhances immune memory	Chen et al., Journal of Controlled Release, 2022. The pullulan/chitosan hydrogel delivered αPDL1 and enhanced the ICD response [185].
			Ye et al., Journal of Controlled Release, 2023. The temperature‐sensitive hydrogel coloaded with α‐CD47 and TMZ enhanced drug delivery and synergistic immune activation [186].
	Facilitating antigen uptake and prolonging duration of action	Enhances antigen presentation, promotes DCs maturation, prolongs antigen release time	Liang et al., Theranostics, 2021. The multifunctional Ncom Gel hydrogel enhanced immunity by efficiently adsorbing antigens and delivering immunomodulatory agonists [204].
			Liu et al., ACS Nano, 2024. The thermosensitive hydrogel enables sustained release of antigens/adjuvants, thereby activating durable immune responses [59].
	Avoiding cytokine storm syndrome	Local sustained release, reduces systemic toxicity, avoids excessive immune activation	Cheng et al., Science Advances, 2023. The thermosensitive hydrogel enables localized retention of R848, CpG, cGAMP, and ICB antibodies, thereby reducing systemic exposure [212].
			Xiong et al., Nickel‐alginate microspheres for sustained IL‐2 release [213].
Small molecules	Enhancing penetration into tumor tissues	Local high concentration, reduces systemic toxicity, enhances T cell infiltration	Ke et al., Advanced Science (Weinh), 2024. The thermosensitive hydrogel enables localized delivery of R848/Zn^2^^+^, sustaining immune cell activation within the TEM [230].
			Li et al., Materials Today Bio, 2022. The codelivery hydrogel continuously and locally delivers DOX/R837 to the TEM [232].
	Enhancing immune‐stimulating capacity	Improves solubility of hydrophobic drugs, prolongs intratumoral retention, activates DCs	Cao et al., Advanced Functional Materials, 2022. The PLGA‐PEG‐PLGA thermosensitive hydrogel achieved high encapsulation efficiency for R837 [239].
			Meng et al., Journal of Controlled Release, 2022. The chitosan/β‐glycerophosphate thermosensitive hydrogel achieved high loading capacity for R837 [240].
	Promoting synergistic immune stimulation	Synergistically activates innate and adaptive immunity, reverses immunosuppression, enhances antitumor effects	Wang et al., Advanced Healthcare Materials, 2023. PVA hydrogel codelivering DMXAA and GEM [244].
			Li et al., Bioactive Materials, 2024. Hyaluronic acid hydrogel delivering RGX‐104 and CD137 agonist [247].
Ions	Enhancing ion delivery	Catalyzes ROS generation, downregulates PD‐L1, induces ICD	Jia et al., Journal of Controlled Release, 2024. Lipoic acid‐Fe^3^^+^ coordination hydrogel inducing ferroptosis [256].
			Shen et al., ACS Nano, 2023. Sodium alginate‐Cu^2^^+^ hydrogel for sustained ES‐Cu release [88].
	Controllable ion release	Maintains local high ion concentration, enhances immune cell infiltration, reduces systemic toxicity	Zhu et al., Science Advances, 2022. The calcium alginate hydrogel ensures effective Ca^2^^+^ retention within the tumor [269].
			Bera et al., Biomaterials Science, 2023. The injectable hydrogel effectively delivers Zn^2^^+^ to the TEM [271].
Nucleic acids/viruses	Facilitating efficient in vivo delivery	High transfection efficiency, local retention, activates T cell response, inhibits tumor growth	Xu et al., International Journal of Pharmaceutical X, 2023. The PEI‐GRAFT‐PEG hydrogel directly encapsulating ABHD5 plasmid [281].
			Wang et al., Angewandte Chemie International Edition Engl, 2024. The nucleic acid hydrogel coloaded with PD‐L1 siRNA and SN38 [108].
	Avoiding enzymatic degradation	Protects nucleic acids from nuclease degradation, prolongs activity, enhances immune response	Jia et al., Advanced Functional Materials, 2022. The alginate hydrogel protecting mRNA‐LNP [284].
			Yin et al., Nano Letters, 2021. The GO‐PEI hydrogel protecting OVA‐mRNA [241].
	Enhancing targeted delivery capabilities	Local retention, reduces liver accumulation, enhances tumor‐specific targeting, reduces systemic toxicity	Du et al., Biomedicine and Pharmacotherapy, 2022. Gelatin hydrogels loaded with oncolytic viruses (OVs) encoding IL‐12 and IL‐15 enhance viral retention specifically at the tumor site [27].
			Lu et al., ACS Nano, 2023. Alginate hydrogels enhance the accumulation of B‐RAF siRNA at the tumor site [296].
	Mitigating virus‐specific immune responses	Reduces neutralizing antibody response, enhances T cell infiltration, prolongs viral action time	Lv et al., Advanced Therapeutics, 2023. Alginate hydrogel reduces the immunogenicity of oncolytic virus (OV) [300].
			Zheng et al., Bioactive Materials, 2021. PLGA‐PEG‐PLGA hydrogel mitigates the acute inflammatory response induced by Sendai virus [301].
Cells	Simulating immune microenvironments	Provides 3D support, prolongs cell survival, enhances cell activity and expansion	Jie et al., ACS Applied Materials & Interfaces, 2022. The self‐assembling peptide‐based hydrogel provides a biomimetic 3D proliferation platform for CAR‐T cells [314]
			Santos et al., Biomaterials Science, 2022. Porous PEG‐heparin hydrogel mimics the architecture of lymphoid tissue [317].
	Promoting local colonization	Local high‐density cell delivery, enhances infiltration, prolongs antitumor immune memory	Hu et al., Nature Biomedical Engineering, 2021. The implantable hyaluronic acid‐based hydrogel directly integrates CAR‐T cells into the tumor bed [327].
			Zhou et al., Biomaterials, 2022. The photocurable GelMA hydrogel enables controlled release of CAR‐T cells at the tumor site [331].
	Acting as an adjuvant	Enhances DCs maturation and antigen presentation, promotes T cell activation	He et al., Biomaterials, 2023. The mannose‐PEI hydrogel encapsulating chemotherapy‐treated tumor cells to enhance immunostimulation [342].
			Yang et al., Acta Biomaterialia, 2021. The cyclodextrin‐polyethylene glycol hydrogel loaded with tumor cell vaccine enhances antigen processing and presentation [139].
Bacteria	Promoting bacterial growth	Provides nutrient microenvironment, selectively promotes beneficial bacteria, enhances immune cell infiltration	Pan et al., Advanced Functional Materials, 2024. The arabinose‐based hydrogel supporting engineered bacteria [366].
			Zheng et al., Nature biomedical engineering, 2022. The mucoadhesive hydrogel promotes the survival of *Peptostreptococcus* [368].
	Improving biocompatibility	Restricts bacterial diffusion, reduces systemic toxicity, promotes local immune activation	Meng et al., Advanced Functional Materials, 2024. Pluronic F127 hydrogel loaded with Microcystis aeruginosa prevents tissue toxicity [375].
			Zhu et al., Biomaterials, 2022. The melittin‐RADA32 hydrogel encapsulating C. novyi‐NT spores enhances bacterial biocompatibility [376].

### 3.1 Hydrogel Containing Protein or Peptide

Protein‐ and peptide‐based therapeutics have emerged as critical components in tumor immunotherapy due to their high specificity, low toxicity, and multifaceted mechanisms of action [155]. This class of agents includes monoclonal antibodies, immune checkpoint inhibitors, cytokines, and targeted peptides, which exert antitumor effects through diverse pathways: activating T‐cell responses, inhibiting immunosuppressive signaling cascades, enhancing antigen presentation, and directly inducing apoptosis in malignant cells [156, 157, 158, 159]. However, these agents face significant challenges in clinical translation, including poor oral bioavailability, short in vivo half‐lives, and the necessity for frequent parenteral administration, which compromise therapeutic sustainability and patient compliance [160, 161]. Additionally, some therapies may trigger excessive immune activation, leading to severe adverse effects such as cytokine release syndrome [162]. In recent years, hydrogels have been developed as innovative drug delivery systems to address these limitations. Their three‐dimensional polymeric networks can encapsulate biologically active macromolecules, enabling sustained release and targeted delivery [163]. Furthermore, functional engineering of hydrogels can enhance immune regulatory capabilities, thereby improving therapeutic efficacy and safety profiles, offering a transformative approach for tumor immunotherapy.

#### 3.1.1 Improving Oral Delivery and Oral Bioavailability

Protein‐ and peptide‐based therapeutics hold a central position in pharmaceutical development due to their cost‐effectiveness, high target affinity, and minimal toxicity. However, their clinical application is hampered by short half‐lives and the need for repeated injections. While the oral route is highly desirable, it presents significant challenges [164]. Hydrogels have emerged as a promising delivery platform that can overcome these hurdles. By physically or chemically encapsulating the cargo, they protect proteins from enzymatic degradation and dilution in the gastrointestinal tract, thereby enabling prolonged systemic retention, enhanced bioavailability, and feasible oral delivery [165].

Selenoproteins is a class of selenium‐containing enzymes crucial for antioxidant defense and immune regulation in the human body, often face limitations in oral delivery due to gastrointestinal degradation and poor absorption. To address stability and absorption barriers in oral protein and peptide delivery, innovative hydrogel systems are being developed [166]. For instance, Ouyang et al. [167] designed an oral hydrogel microsphere system using calcium alginate to encapsulate hyaluronic acid‐modified selenium nanoparticles. This system enables the in situ synthesis of selenoproteins in the intestine, effectively bypassing the challenges associated with delivering intact proteins and significantly enhancing their oral bioavailability. Beyond improving delivery efficiency, the microsphere treatment also demonstrated immunomodulatory potential by markedly reducing the secretion of proinflammatory cytokines, decreasing neutrophil and monocyte counts, and increasing regulatory T cell populations.

Hydrogels improve mucosal immunity and antigen presentation in oral vaccines, which can address the compliance issues associated with multidose injectable regimens [168, 169]. For example, Chen et al. [170] developed bacterial nanocellulose/polyacrylic acid hydrogel microparticles (BNC/PAA MPs) for oral delivery of hepatitis B surface antigen. These MPs enhanced oral bioavailability via pH‐responsive protection and mucoadhesion, and elicited intestinal sIgA and serum IgG antibodies in mice. They also activated CD4^+^ T cells and CD19^+^ B cells, demonstrating effective lymphocyte priming.

Exendin‐4 is a glucagon‐like peptide‐1 (GLP‐1) receptor agonist whose oral administration confronts the common challenge of extremely low bioavailability shared by many peptide drugs [171]. Utilizing a hydrogel‐based delivery system to protect the peptide from gastrointestinal degradation and enhance its intestinal permeability presents a key strategy for improving its oral bioavailability. Zhou et al. [172] developed an oral peptide delivery system utilizing a synergistic combination of a zwitterionic hydrogel‐coated MOF nanoparticle and a pH‐triggered capsule. The pH‐sensitive capsule shell ensures the carrier remains intact in the acidic gastric environment and rapidly disintegrates in the neutral pH of the intestine, while the zwitterionic hydrogel layer provides antimucoadhesive properties. These properties collectively enhance the oral bioavailability of exendin‐4 and demonstrate remarkable therapeutic efficacy in animal models.

Hydrogels also enable oral delivery of immune checkpoint inhibitor peptides to reverse T‐cell exhaustion. For instance, Li et al. [173] developed an N,N,N,N‐trimethyl CS (TMC) hydrogel to encapsulate OPBP‐1, a d‐peptide that selectively blocks PD‐1/PD‐L1 interaction [174]. The TMC hydrogel provided sustained release under gastrointestinal conditions and extended the half‐life of OPBP‐1 in rats to 14.55 h, 44.1‐fold longer than intravenous administration. This study represents the first successful oral delivery of a PD‐1/PD‐L1‐targeting peptide, which systemically blocks T‐cell exhaustion signals and reactivates cytotoxic T‐cell function in the TME. Although the gastrointestinal environment poses unique challenges for oral protein and peptide therapeutics, advances in hydrogel technology, coupled with the growing clinical relevance of peptide drugs, suggest that innovative delivery systems capable of overcoming intestinal barriers will continue to emerge, thereby expanding the clinical applications of polypeptide‐based therapies.

#### 3.1.2 Augment Immune Responses

Hydrogel‐based delivery systems have emerged as a powerful platform to enhance antitumor immunity by overcoming the pharmacokinetic limitations of immunomodulatory agents and enabling spatiotemporally controlled release within the TME [175, 176]. Localized delivery of immune checkpoint blockers via hydrogels can minimize systemic exposure while enhancing therapeutic efficacy. Chen et al. [177] developed an injectable pullulan/CS hydrogel that serves as an immunoenhancing platform for synergistic immunotherapy. By sustaining αPDL1 antibody release and inducing ICD, the system promotes DC maturation and locally blocks PD‐1/PD‐L1 to activate cytotoxic T cells. This enhanced immunity led to robust T cell infiltration in both primary and distant tumors and reversal of the immunosuppressive microenvironment, enabling effective postoperative treatment.

In glioma treatment, Ye et al. [178] developed a thermosensitive hydrogel (α‐CD47 and TMZ@Gel) coloaded with an α‐CD47 antibody and temozolomide for local implantation into the postsurgical cavity. The hydrogel's three‐dimensional network enables dual‐signal regulation that activates macrophages and subsequently recruits and activates cytotoxic T cells and natural killer (NK) cells. Furthermore, by ensuring localized drug release within the surgical site, the system promotes the secretion of proinflammatory cytokines such as TNF‐α and IFN‐γ, thereby reversing the immunosuppressive state and significantly suppressing tumor recurrence. Cytokines such as interleukin (IL)‐12, IFN‐γ, and IL‐15 are potent immune activators, yet their clinical application is limited by short half‐lives and rapid systemic clearance [179, 180]. By providing a microenvironment for localized retention and controlled release, hydrogel technology effectively overcomes these pharmacokinetic limitations and significantly enhances the immunostimulatory capacity of cytokines [181]. For instance, Lee et al. [182] developed a bilayer adhesive nanofibrous hydrogel for the temporal release of IL‐12 and IFN‐γ in combination with the chemotherapeutic agent DOX. This system not only induced ICD to release tumor antigens but also leveraged the sustained release properties of the hydrogel to maintain effective local concentrations of IL‐12 and IFN‐γ. This synergistic action promoted DC maturation and T‐cell activation, ultimately eliciting potent antitumor immunity. In a complementary approach, Grosskopf et al. [183] utilized an injectable polymer–nanoparticle (PNP) hydrogel for the codelivery of IL‐15 and CAR‐T cells. The hydrogel effectively retained IL‐15 via hydrophobic interactions, significantly prolonging its local presence and creating a transient “inflammatory niche” in vivo. This microenvironment markedly enhanced the proliferation, persistence, and tumor‐responsive activity of CAR‐T cells, demonstrating the considerable potential of hydrogels in optimizing cytokine‐mediated cell therapies.

Hydrogel‐based delivery systems significantly enhance the immunostimulatory efficacy of immunomodulatory peptides by improving their pharmacokinetic profiles and spatiotemporal distribution [184]. For instance, Zhang et al. [185] constructed an injectable peptide hydrogel (BV/TP5) that, under near‐infrared irradiation, not only disrupted the tumor extracellular matrix and alleviated hypoxia but also acted synergistically with the immunomodulatory peptide thymopentin (TP5) to reverse local immunosuppression. This approach markedly promoted T‐cell infiltration, thereby converting immunologically “cold” tumors into “hot” tumors. DPPA‐1 is a d‐peptide antagonist with high affinity for PD‐L1, functions by locally blocking the PD‐1/PD‐L1 immune checkpoint to augment T‐cell‐mediated antitumor responses [186]. In another synergistic strategy, Liu et al. [187] developed a supramolecular hydrogel for the codelivery of DOX and the PD‐L1 antagonist peptide DPPA‐1. This system rapidly releases DOX to induce ICD and release tumor‐associated antigens, while sustainably releasing the DPPA‐1 peptide to block PD‐1/PD‐L1 interactions. Together, these actions synergistically activate T cells and enhance their tumor‐infiltration capacity, significantly improving the antitumor efficacy of the combination therapy. Collectively, these studies demonstrate that hydrogels not only optimize the pharmacokinetic behavior of peptide drugs but also effectively enhance antitumor immune responses through temporal release control and local microenvironment modulation. Therefore, hydrogel delivery systems significantly enhance the efficacy and clinical translation potential of tumor immunotherapy by overcoming the pharmacokinetic limitations of immunomodulatory peptides and synergizing multiple therapeutic mechanisms [9, 188].

#### 3.1.3 Facilitate Antigen Uptake and Prolong Duration of Action

Antigens are crucial for inducing specific immune responses, particularly through antibody production [189]. The magnitude of these responses is highly dependent on antigen properties such as size, structure, and composition, as well as the efficiency of antigen uptake by immune cells [190]. Hydrogels, serving as effective delivery vehicles, enhance antigen immunogenicity by improving uptake efficiency and enabling controlled, sustained release. Their biocompatible nature and ability to incorporate immunomodulatory agents allow them to attract immune cells to the injection site and amplify immune activation [191, 192, 193].

**FIGURE 3:**
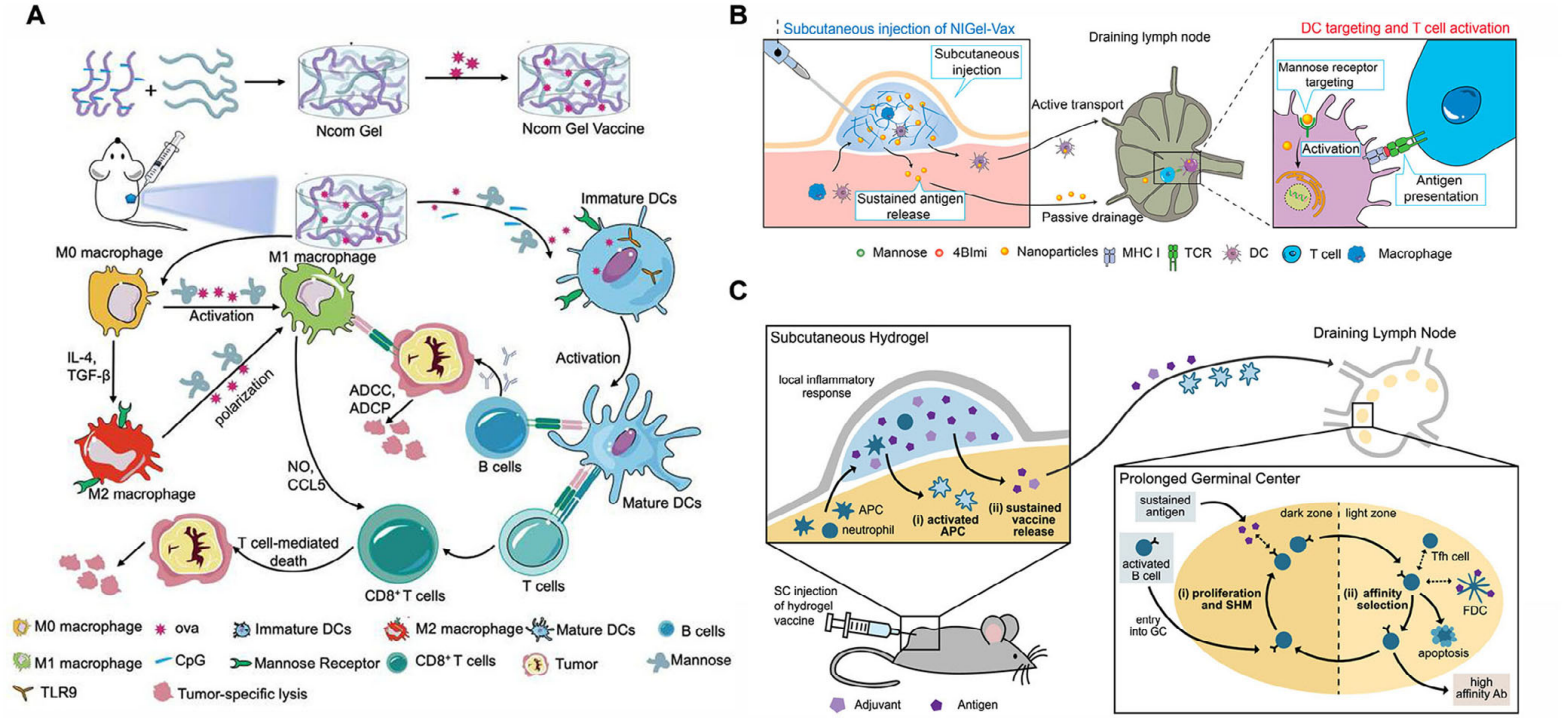
(A) The self‐assembling NOCC─CpG/OX‐M hydrogel vaccine (Ncom Gel) was engineered to mobilize antitumor innate immunity. Administration of Ncom Gel enhanced innate immune activity via DCs and macrophage activation. Critically, this platform also enabled innate immune cells to initiate and sustain robust adaptive immune responses against tumors. Reprinted with permission from Ref. [196], Copyright 2021 Lvyspring International Publisher. (B) The NIGel‐Vax platform integrates protein antigens with PEI‐4BImi‐Man via spontaneous nanoparticle self‐assembly, subsequently encapsulated within an injectable hydrogel matrix. Following subcutaneous administration, the hydrogel depot enables sustained nanoparticle release and lymphatic trafficking to draining lymph nodes. There, DCs internalize nanoparticles, driving crosspresentation of antigens to T cells and eliciting antigen‐specific adaptive immunity. Reprinted with permission from Ref. [55], Copyright 2020 American Chemical Society. (C) In vivo response of the PNP hydrogel to prolonged hydrogel‐based vaccine delivery. The hydrogel depot recruits neutrophils and antigen‐presenting cells (APCs), inducing localized activation. Critically, two synergistic mechanisms drive immunity: (i) activated APCs traffic to draining lymph nodes (dLNs) for T‐cell priming; (ii) sustained antigen/adjuvant release from the hydrogel prolongs germinal center (GC) activity in dLNs. Reprinted with permission from Ref. [199], Copyright 2020 American Chemical Society.

A primary strategy involves using hydrogels as scaffolds to codeliver antigens and adjuvants, thereby enhancing antigen presentation and immune stimulation [194, 195]. Antigen‐only formulations often elicit suboptimal T‐cell responses compared with live‐attenuated or viral vector vaccines [193]. To overcome this limitation, Liang et al. [196] developed a self‐assembled, multifunctional NOCC─CpG/OX‐M hydrogel (Ncom Gel) vaccine with a highly crosslinked, porous structure. This design facilitates antigen adsorption and the loading of mannose receptors and Toll‐like receptor 9 (TLR9) agonists. The hydrogel protects immunomodulatory components from degradation while preserving antigen integrity. Experimentally, it significantly increased antigen uptake by DC2.4 cells and promoted the secretion of proinflammatory cytokines, thereby synergistically enhancing both innate and adaptive immune responses (Figure 3). Hydrogels also serve as platforms for postsurgical immunotherapy to prevent tumor recurrence. Tumor antigen vaccines represent a promising strategy for improving disease‐free survival by eliciting antigen‐specific immune responses [197]. Liu et al. [55] constructed an injectable nanoparticle‐in‐gel vaccine, NIGel‐Vax, in which the hydrogel system facilitates active uptake of antigens by DCs through its loaded functionalized nanoparticles. Simultaneously, the tunable degradable hydrogel matrix acts as a local depot, enabling sustained antigen release and significantly prolonging the duration of vaccine action. NIGel‐Vax elicits robust antigen‐specific humoral and cellular immunity, markedly enhances the infiltration of effector T cells into tumor sites, and ultimately induces a durable antitumor immune response (Figure 3). One key challenge faced by cancer vaccines is their unsatisfactory immunogenicity, which stems from the need for continuous antigenic stimulation to achieve long‐term efficacy. Many existing antigenic vaccines lack suitable carriers or adjuvants to provide this prolonged exposure [198]. Roth et al. [199] engineered a sustained‐release vaccine platform using an injectable, self‐repairing PNP hydrogel. This platform prolongs the codelivery of vaccine components to immune cells. In murine models, the sustained antigen exposure significantly enhanced the magnitude, duration, and quality of humoral immune responses. It amplified the germinal center response in lymph nodes, promoted antibody affinity maturation, and resulted in a more than 1000‐fold increase in antigen‐specific antibody affinity compared with conventional bolus immunizations (Figure 3).

Furthermore, hydrogels show great potential in the field of personalized tumor vaccines. The efficacy of antigenic drugs relies on efficient uptake and sustained bioactivity. Hydrogels, with their tunable physicochemical properties, optimize these aspects [176]. For instance, Phuengkham et al. [200] constructed a hydrogel termed immuneCare‐DISC (iCD), composed of collagen and hyaluronic acid. As a three‐dimensional porous scaffold, it was loaded with whole tumor lysates as an antigen source, effectively recruiting DCs into its porous network. Simultaneously, the iCD was designed as a sustained‐release system capable of continuously releasing antigens and adjuvants over several weeks, activating DCs and macrophages, which in turn activated antigen‐specific CD4^+^ and CD8^+^ T cells. Ultimately, this approach successfully elicited a potent systemic antitumor immune response, effectively preventing postoperative tumor recurrence and metastasis.

In summary, hydrogels leverage their unique material properties, such as porous structures for codelivery, injectability for localized deployment, and tunable release kinetic to facilitate antigen uptake and prolong immunostimulation. They thereby address key limitations in current vaccine strategies and provide a versatile platform for advancing cancer immunotherapy.

#### 3.1.4 Avoid Cytokine Storm Syndrome

Protein/peptide immunostimulants constitute pivotal tools in tumor immunotherapy by activating specific immune responses [159]. However, their clinical translation requires rigorous safety evaluation [201]. These biologics can elicit immunogenicity, leading to antidrug antibodies that compromise efficacy and cause adverse effects including hypersensitivity. More critically, while immune activation underpins their therapeutic mechanism, excessive stimulation may provoke pathological responses, particularly cytokine release syndrome. This life‐threatening complication limits systemic cytokine therapy, but local administration based on hydrogels holds promise in avoiding this risk [202, 203].

To address these challenges, Cheng et al. [204] developed an injectable thermoresponsive hydrogel (NvIH) that integrates triple‐immunostimulant nanoparticles and immune checkpoint blockers. This platform enables localized retention and sustained release of therapeutic agents at the tumor site, thereby enhancing the durability of antitumor immune responses while minimizing systemic exposure. Safety assessments demonstrated significantly lower serum levels of key inflammatory cytokines, including IL‐6, IL‐12p70, IP‐10, MIP‐1β, and MIG in the NvIH group compared with nonhydrogel controls at 4 h posttreatment. These findings confirm that the hydrogel formulation effectively restricts systemic cytokine dissemination, substantially improving treatment tolerability and mitigating the risk of cytokine storm.

Complementing this approach, Xiong et al. [205] engineered nickel‐alginate hydrogel microspheres (Ni‐ALGMS) for efficient IL‐2 loading and sustained release. This innovative system achieves selective cytokine binding through coordination between alginate carboxyl groups and Ni^2^^+^ ions, resulting in significantly higher drug loading capacity compared with conventional Ca^2^^+^‐crosslinked alginate microspheres. The Ni‐ALGMS platform substantially prolongs IL‐2 retention within tumors, effectively enhancing T lymphocyte infiltration and activation while preventing systemic cytokine dissemination. Notably, this approach successfully mitigates cytokine storm risk without observed Ni^2^^+^‐related toxicity, validating its potential as a safe localized cytokine delivery strategy.

Collectively, these hydrogel‐based systems demonstrate that localized, sustained release of immunostimulatory agents can effectively uncouple therapeutic efficacy from systemic toxicity. By maintaining high local drug concentrations while minimizing systemic exposure, hydrogels provide a robust technological solution to mitigate cytokine storm syndrome.

### 3.2 Hydrogel Containing Small Molecules

Small molecule drugs are defined as organic compounds with molecular weights typically below 1000 Da [206]. These compounds exert therapeutic effects by modulating biological processes, thereby aiding in the diagnosis, treatment, or prevention of diseases. Although small molecule drugs lack the specificity of antibodies and cytokines, they offer several advantages, including straightforward synthesis [207], cost effectiveness, high oral bioavailability [208], and the ability to traverse cell membranes [209]. In tumor immunotherapy, these agents enhance immunotherapeutic outcomes through multiple mechanisms, such as immune checkpoint inhibition [210], immune modulation [211], and the activation or suppression of signaling pathways [212]. However, the rapid systemic diffusion of small molecule drugs necessitates frequent administration. This limitation can be addressed through hydrogel‐based delivery systems. Hydrogels, capable of retaining large volumes of liquid without dissolving [213], provide protection and controlled release for small molecule drugs. Additionally, when employed as a local delivery vehicle, hydrogels enhance drug permeability in tissues such as the skin or mucous membranes, improving bioavailability and promoting targeted tumor cell delivery [214]. Simultaneously, this localized approach reduces systemic toxicity, offering a safe and efficient strategy for therapeutic intervention.

#### 3.2.1 Enhance Penetration Into Tumor Tissues

Small molecule immunotherapeutics face significant clinical challenges due to their propensity for systemic distribution and off‐target effects when administered orally or intravenously [215, 216, 217]. These limitations often result in severe adverse effects including myelosuppression, gastrointestinal disturbances, and cardiotoxicity, which substantially restrict their therapeutic window [218, 219].

The biocompatible and minimally irritating nature of natural and composite hydrogels enables direct injection into tumor sites, effectively confining drug exposure to target areas while minimizing systemic dissemination [214]. This localized approach significantly enhances the therapeutic index of potent immunotherapeutic agents [220]. In a compelling demonstration, Ke et al. [221] developed a thermosensitive hydrogel (FSP‐RZ‐BPH) for codelivering R848 and Zn^2^^+^, which reduced serum levels of inflammatory cytokines IL‐6 and TNF‐α to one‐third of those observed with subcutaneous administration. This localized delivery system maintained sustained DC activation in the TME and achieved a 2.5‐fold increase in CD8^+^ T cell infiltration in draining lymph nodes, confirming precise spatiotemporal control over immune agonist release. Serving as local drug depots, hydrogels can effectively enhance the penetration and retention of small‐molecule drugs within tumor tissues [222]. In the DOX and imiquimod (R837) codelivery hydrogel system developed by Li et al. [223], the drugs are continuously and locally delivered to the TME, overcoming the limitations of conventional intravenous administration such as poor tumor penetration and widespread systemic distribution. This hydrogel‐mediated deep penetration and synergistic action triggered a potent antitumor immune response, resulting in a 4.2‐fold increase in tumor‐infiltrating CD103^+^ DCs and the expansion of antigen‐specific CD8^+^ T cells. Concurrently, the plasma drug concentration was only 1/15 of that achieved with intravenous administration, and no cardiotoxicity was detected. This fully demonstrates that by enhancing drug penetration and retention in tumor tissues, hydrogels can maximize the synergistic therapeutic efficacy while significantly reducing systemic toxicity.

Beyond simple drug delivery, hydrogels can fundamentally reshape the tumor immune microenvironment through controlled release kinetics. Wu et al. [224] encapsulated the TLR7/8 agonist IMDQ in a dynamically crosslinked hydrogel, which controlled the peak serum IL‐6 concentration within a safe window and maintained a favorable immune contexture in the TME characterized by an M1 macrophage‐to‐regulatory T cell ratio exceeding 8:1. Similarly, Xiao et al. [225] employed an alginate‐based hydrogel for R848 delivery, which persistently activated the IRF5 signaling pathway in macrophages, resulting in a 68.5% conversion rate from M2 to M1 phenotype. This approach significantly alleviated the immunosuppressive microenvironment without causing histopathological damage in vital organs.

Collectively, these studies establish that hydrogel technology enhances tumor tissue penetration through three interconnected mechanisms: spatiotemporal control of drug release, optimization of immune cell interactions, and active remodeling of the TME. This multifaceted approach provides an innovative strategy to overcome the fundamental limitations of small‐molecule immunotherapeutics, offering a pathway to enhanced efficacy without compromising safety.

#### 3.2.2 Enhance Immune‐Stimulating Capacity

A subset of small molecule drugs exhibits hydrophobic characteristics, including low solubility, poor absorption, high plasma protein binding, and impaired metabolic stability [226]. These properties pose significant challenges during the absorption, distribution, metabolism, and excretion processes, directly impacting therapeutic efficacy and safety. In the context of tumor immunotherapy, the bioavailability of hydrophobic small molecule drugs remains a critical area for improvement. The emergence of hydrogels has revolutionized the delivery and therapeutic application of small molecule drugs in recent years [227]. These hydrogels leverage their hydrophilic nature to create a supportive microenvironment for hydrophobic drugs, thereby improving drug encapsulation efficiency [228]. Furthermore, modulating the physicochemical properties of hydrogels, such as crosslinking density or pore architecture, can enhance the solubility of hydrophobic compounds.

The hydrophilic polymer networks of hydrogels can effectively encapsulate hydrophobic drug molecules and improve their dispersion through hydrogen bonding, van der Waals forces, and hydrophobic interactions [229]. Cao et al. [230] developed a PLGA–PEG–PLGA thermosensitive hydrogel coloaded with the adjuvant R837 and calcium salts. This system rapidly releases Ca^2^^+^ to induce ICD in tumor cells, while utilizing CaCO_3_ to modulate the microenvironmental pH for sustained R837 release. This approach significantly enhanced the infiltration of CD4^+^/CD8^+^ T cells into tumors and promoted the formation of memory T cells. Similarly, alginate–Ca^2^^+^‐based hybrid RA gels achieved nearly 100% encapsulation efficiency for R837, maintaining homogeneous dispersion in acidic solutions and effectively promoting DC maturation and T‐cell activation via TLR7/8 signaling pathway activation.

Preformulating hydrophobic drugs into nanocrystals before incorporating them into hydrogels can further enhance drug loading capacity and stability. In a specific study, Meng et al. [231] constructed a thermosensitive CS/β‐glycerophosphate‐based hydrogel encapsulating polydopamine‐coated R837 nanocrystals. This system not only achieved high loading and prolonged intratumoral retention of the hydrophobic immunoadjuvant but also sustained DC activation through spatiotemporally controlled release. When combined with mild PTT, it increased the proportion of CD8^+^ T cells to 15.9% and significantly suppressed melanoma growth and metastasis. This system leveraged R837's alkaline nature and accelerated dissolution under acidic conditions to achieve pH‐responsive release within the TME.

Introducing hydrophobic functional groups into hydrophilic hydrogels significantly enhances drug–material interactions, addressing the issues of low loading efficiency and drug leakage associated with conventional hydrophilic systems. For instance, Yin et al. [232] constructed a hydrogel via electrostatic interactions between low‐molecular‐weight polyethylenimine and graphene oxide (GO) for R848 delivery. The strong π–π stacking between R848 and GO achieved 97% encapsulation efficiency. This delivery system mediated nearly 100% penetration of CD8^+^ IFN‐γ^+^ T cells in the TME, significantly outperforming the 50% achieved with R848 suspensions, demonstrating that hydrogels can effectively improve the compatibility of hydrophobic drugs with the TME.

In summary, through their tunable physicochemical properties, nano‐integration strategies, and hydrophobic modification technologies, hydrogels effectively address the challenges of solubility, stability, and controlled release associated with hydrophobic immunoadjuvants. This thus provides multiple reliable technological pathways for enhancing antitumor immune responses.

#### 3.2.3 Promote Synergistic Immune Stimulation

The mechanism of action for small molecule drugs is typically well defined, as these agents often target specific biological pathways or molecular entities. However, in complex or multifactorial diseases such as cancer, monotherapies may fail to achieve optimal therapeutic outcomes due to insufficient control of the disease or inadequate efficacy [233]. In such cases, combination therapy emerges as a more comprehensive strategy, and hydrogels—functioning as versatile drug carriers—can facilitate this approach. Hydrogels enable the integration of small molecule drugs with distinct mechanisms of action, generating synergistic effects. Moreover, they can be engineered as smart materials responsive to external stimuli, ensuring precise and controlled release of multiple therapeutic agents [234]. This synergistic potential is exemplified by the combined activation of innate and adaptive immunity, as demonstrated with STING agonists and chemotherapy. Specifically, DMXAA activates the STING pathway in antigen‐presenting cells (APCs) to promote their maturation and enhance T‐cell responses, but it can also induce indoleamine 2,3‐dioxygenase. To counter this and amplify immunogenicity, Wang et al. [235] developed a PVA hydrogel to codeliver DMXAA and gemcitabine (GEM). While GEM induces ICD to release tumor antigens, DMXAA enhances DC antigen uptake and crosspresentation via STING activation. This combination, facilitated by the hydrogel, dramatically increased tumor‐infiltrating CD8^+^ T cells to 47.4%, far exceeding monotherapies, achieving potent efficacy with lower systemic drug exposure.

A further refinement involves tailoring drug release kinetics to coordinate sequential immune interventions, such as reversing immunosuppression before activating T‐cells. RGX‐104, a small molecule agonist of the liver X nuclear receptor beta (LXR‐β), activates the LXR‐β/apolipoprotein E axis to inhibit myeloid‐derived suppressor cell (MDSC) survival and reduce their abundance [236]. However, monotherapy with RGX‐104 may yield limited clinical benefits due to insufficient immune modulation [237]. To address this, Li et al. [238] utilized a hyaluronic acid hydrogel for the staggered codelivery of RGX‐104, an LXR‐β agonist, and a CD137 agonist antibody. The system was designed for rapid initial release of RGX‐104 to inhibit MDSC survival and reduce IL‐10 levels, thereby mitigating local immunosuppression. This was followed by the sustained release of the CD137 agonist, which promoted T‐cell proliferation and effector functions within the newly permissive immune microenvironment, orchestrating a coordinated transition from immune escape to elimination.

Finally, localized codelivery systems can synergistically enhance antigen presentation and block immune checkpoints directly within the TME. Combination immunotherapy has demonstrated significant advantages in tumor treatment by enhancing the immune system's capacity to recognize and eradicate malignant cells. However, this approach faces two major challenges, only approximately 20–30% of patients respond to single‐agent or combination therapies, often due to tumor heterogeneity or immunosuppressive microenvironments. And systemic drug exposure may lead to severe adverse effects. To overcome these limitations, precision drug delivery systems are being explored to optimize immunotherapeutic strategies. Specifically, hydrogels serve as multidrug carriers, enabling the synergistic delivery of immune activators and chemotherapeutics. Localized drug release within the TME enhances immune response specificity and persistence while minimizing systemic toxicity and sustaining immune activation. Gu et al. [52] developed an in situ‐forming fibroin–CS composite scaffold to regulate the corelease of DOX and JQ1, a BET inhibitor. This combination works by having DOX induce ICD to release tumor antigens and promote DC maturation, while JQ1 simultaneously downregulates PD‐L1 expression on tumor cells via BRD4 inhibition, effectively blocking the PD‐1/PD‐L1 checkpoint to prevent T‐cell exhaustion. Similarly, Mei et al. [235] engineered a thermoresponsive CS hydrogel to coencapsulate indocyanine green and imiquimod, which, combined with cyclophosphamide, enhanced immune activation. By confining the action of such synergistic combinations locally, hydrogel platforms enhance the specificity and persistence of antitumor immunity while minimizing systemic toxicity, offering a promising pathway for more effective and tolerable cancer therapies.

### 3.3 Hydrogel Containing Ions

Trace element ions, including ion (Fe^2^^+^/Fe^3^^+^), manganese (Mn^2^^+^), copper (Cu^2^^+^), and magnesium (Mg^2^^+^), play essential roles in human physiology. These ions serve as cofactors for numerous enzymes, which are critical for maintaining optimal immune function and antioxidant defense mechanisms [239]. Beyond their fundamental physiological roles, trace elements contribute significantly to immune regulation, particularly in tumor immunotherapy [240]. The administration of specific trace elements has been demonstrated to induce tumor cell apoptosis and enhance immune system activation, thereby improving therapeutic outcomes [241]. However, the direct systemic delivery of these ions in ionic form presents challenges due to their limited bioavailability and the necessity for precise concentration control to achieve therapeutic efficacy [242]. In this context, hydrogels have emerged as promising platforms for ion delivery systems. Encapsulation of trace element ions within hydrogels enables controlled release and sustained concentration maintenance, mitigating the risks associated with excessive or insufficient local concentrations that may occur with direct injection [243, 244]. The unique properties of hydrogels—such as biocompatibility, tunable degradation rates, and responsive behavior—facilitate the establishment of a continuous ion release environment while ensuring biosafety and therapeutic effectiveness at the target site [245].

#### 3.3.1 Enhance Ions Delivery

Copper (Cu^2^^+^), zinc (Zn^2+^), and iron (Fe^2^^+^/Fe^3^^+^) ions have been implicated in programmed cell death pathways characterized by distinct metabolic alterations. These ions induce cancer cell death through multiple mechanisms, representing novel modalities of cell death beyond apoptosis or necrosis [246]. The efficacy of these metal ions is strictly dose dependent, requiring sustained elevated intracellular concentrations to effectively initiate cell death pathways [247]. Consequently, therapeutic interventions often necessitate carriers to achieve the necessary local concentrations while minimizing systemic toxicity. Hydrogels have emerged as promising platforms capable of inducing tumor‐specific cell death while preserving biosafety. For instance, Jia et al. [248] developed a lipoic acid–Fe^3^^+^ coordination hydrogel (LFH) that effectively induces ferroptosis—an iron‐dependent form of regulated cell death characterized by lipid peroxidation. The gradual degradation of LFH promotes the reduction of Fe^3^^+^ to Fe^2^^+^, which catalyzes the Fenton reaction to generate abundant ROS. In GL261 cells, the ROS level in the LFH‐treated group (1587%) was 2.8‐fold and 2.0‐fold higher than in the Fe^3^^+^ group (573%) and LA/LANa group (785%), respectively. More importantly, this ferroptotic cell death is immunogenic, as evidenced by significantly elevated ATP secretion and calreticulin exposure, which collectively promote DC maturation and activate antitumor T‐cell responses, thereby amplifying Fe^3^^+^‐mediated ICD.

**FIGURE 4:**
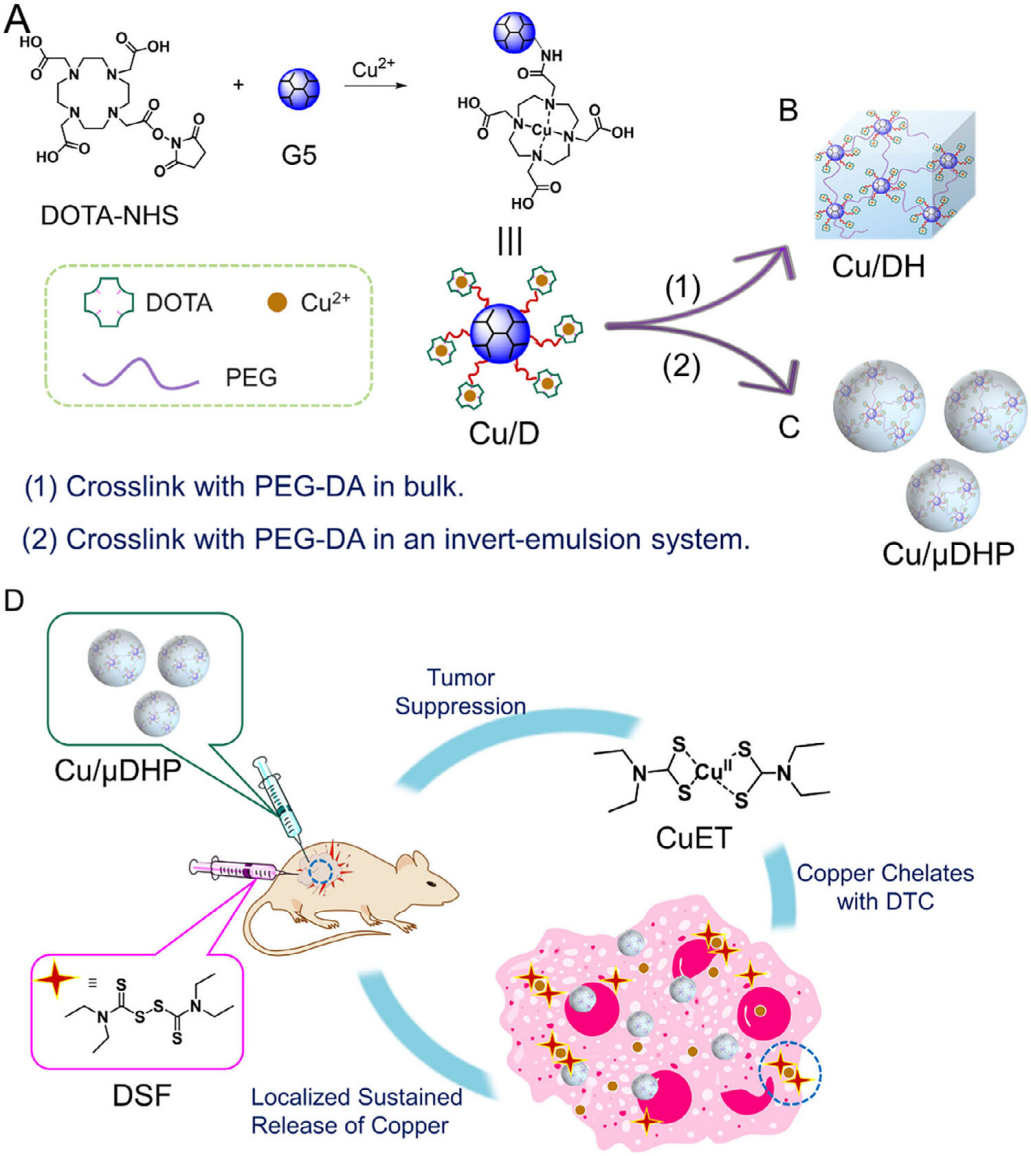
Schematic illustration of (A) the synthesis of G5‐DOTA and Cu/D, (B and C) the preparation of Cu/DH and Cu/μDHP, (D) intratumoral injection of Cu/μDHP and DSF for the treatment of HN12 tumor‐bearing mouse. Reprinted with permission from Ref. [252], Copyright 2024 American Chemical Society.

Hydrogels represent multifunctional biomaterials with distinct advantages in drug delivery systems. By precisely modulating degradation rates and pore architecture, hydrogel drug release profiles can be engineered to achieve sustained and controlled delivery, which is particularly critical for ionic therapeutics to maintain stable concentrations over extended periods [249]. Furthermore, hydrogels can be designed as stimuli‐responsive materials capable of autonomously adjusting drug release in response to physiological conditions such as pH, temperature, or enzymatic activity, ensuring localized delivery contingent on specific microenvironmental cues [250, 251]. For instance, Shen et al. [84] developed prefabricated hydrogels by conjugating isomers (ES) to Cu^2^^+^ ions on sodium alginate chains. When placed in an acidic TME (pH 5.6), the hydrogels sustained ES‐Cu release over approximately ten days. This prolonged release not only maintains Cu^2^^+^ concentrations within the therapeutic window but also induces copper‐dependent PD‐L1 downregulation in tumor cells, thereby blocking the PD‐1/PD‐L1 immune checkpoint and enhancing T‐cell‐mediated tumor killing, while simultaneously avoiding systemic toxicity associated with high‐dose elecomox or Cu^2^^+^ administration. In another approach, Wang et al. [252] engineered micron‐scale hydrogel particles (Cu/μDHP) based on polyamidoamine (PAMAM) dendritic polymers, which enabled slow Cu^2^^+^ release sustained for up to 144 h. This controlled release mechanism ensures adequate Cu^2^^+^ levels to initiate antitumor therapy while mitigating toxicity risks—when Cu^2^^+^ levels increased from 4.4 to 44 µM, the cell death rate in nonhydrogel groups exceeded 50%, whereas only 28% of cells in the hydrogel group exhibited mortality (Figure 4). The crosslinked structure of hydrogels imparts inherent slow‐release properties [253]. By adjusting hydrogel crosslinking density and architecture, the release rate can be optimized to ensure continuous, stable delivery at the target site, preserving the desired therapeutic window [140].

#### 3.3.2 Controllable Ion Release

The in vivo delivery of ionic drugs presents unique challenges, primarily due to their physicochemical properties and the dynamic in vivo environment [254]. Most ionic drugs carry a net positive or negative charge, a characteristic that facilitates interactions with biological structures such as cell membranes but simultaneously increases resistance to cellular penetration [255]. Additionally, the high water solubility of many ionic drugs promotes rapid dispersion in the bloodstream, hindering the maintenance of effective therapeutic concentrations at target sites. Addressing these limitations necessitates strategies to enhance drug targeting and stability [256]. In this context, hydrogels, as advanced biomaterials, offer a promising solution for the controlled delivery of ionic drugs. The integration of ionic drugs with hydrogels to form chelates has been shown to improve drug stability and enable precise regulation of release kinetics [257]. Hydrogels can be engineered to respond to internal structural modifications or external stimuli—such as pH, temperature, or enzymatic activity—allowing for tunable drug release rates. This adaptability enhances bioavailability, minimizes adverse effects, and reduces the frequency of dosing by maintaining stable drug concentrations over prolonged periods [258, 259]. Furthermore, the programmable nature of hydrogel‐based systems enables intelligent, site‐specific drug delivery tailored to diverse therapeutic requirements [260]. Zhu et al. [261] demonstrated a novel approach to encapsulating calcium ions (Ca^2^^+^) within sodium alginate to form a calcium alginate hydrogel (Ca^2^^+^ hydrogel). Upon intratumoral injection, the metallic alginate gel functions as a microwave‐sensitive agent, amplifying the thermal ablation of primary tumors. Elevated extracellular Ca^2^^+^ disrupts intracellular calcium homeostasis in cancer cells, rendering them susceptible to mild heat‐induced damage. This mechanism enables complete tumor ablation within 24 h postinjection. In the presence of alginate hydrogels, approximately 53.1% of Ca^2^^+^ remained localized at the tumor site, compared with only 26.8% retention for free Ca^2^^+^ in the absence of hydrogel encapsulation. The superior ion‐chelating capacity of hydrogels ensures effective Ca^2^^+^ retention in tumors, thereby enhancing the synergistic efficacy of microwave ablation. By leveraging chelation technology, ions with specific biological functions can be selectively integrated into hydrogels to create slow‐release systems. These systems sustain therapeutic concentrations in target regions, such as the TME, while minimizing systemic exposure. This localized delivery strategy not only prolongs drug action but also significantly reduces toxicity to nontarget tissues and systemic side effects. Such approaches represent a paradigm shift in precision medicine, offering improved therapeutic outcomes and enhanced patient quality of life. Zinc ions (Zn^2^^+^) play a critical role in modulating immune responses by enhancing the catalytic activity of cyclic GMP–AMP synthase (cGAS) and its capacity to recognize damaged DNA. Activation of the cGAS/STING signaling pathway upregulates type I interferon and proinflammatory cytokines, thereby stimulating antitumor immunity [262]. Bera et al. [263] developed a drug‐free hydrogel for melanoma immunotherapy by coordinating Zn^2^^+^ with 5‐amino‐1,10‐phenanthroline and melofenac sodium. The resulting hydrogel exhibited injectable properties and effectively delivered Zn^2^^+^ to the TME. Compared with melofenac sodium alone (10%), the hydrogel significantly increased CD8^+^ T cell infiltration (60%) and elevated levels of anti‐inflammatory mediators (IFN‐γ, IL‐2, IL‐6, and TNF‐α). Jin et al. [264] further advanced this concept by designing a STING‐activated hydrogel (ZCCG) through the coordination of Zn^2^^+^ with 4,5‐imidazole dicarboxylic acid (IDA). Postdelivery, the sustained release of Zn^2^^+^ enhanced cGAS enzymatic activity, thereby potentiating STING pathway activation. These findings underscore the versatility of hydrogel platforms in harnessing ion‐specific biological functions for targeted immunotherapy.

### 3.4 Hydrogel Containing Nucleic Acids or Viruses

Nucleic acid and viral drugs represent a transformative application of modern biotechnology in medicine. Nucleic acid‐based therapies employ DNA or RNA molecules, such as small interfering RNA (siRNA) and antisense oligonucleotides, to modulate gene expression or correct genetic defects [265]. Viral vectors, engineered through genetic modification, selectively target tumor cells while stimulating immune responses against malignancies [266]. Adenoviral vectors are widely utilized in tumor immunotherapy, where they deliver functional genes into patient cells to compensate for defective genetic pathways or achieve therapeutic objectives [267]. Hydrogels, as advanced biomaterials, offer dual advantages in this context: they protect nucleic acid drugs from enzymatic degradation and enable targeted release, thereby enhancing therapeutic precision. For adenoviral therapies, hydrogels mitigate virus‐specific immune responses, improving safety profiles. Consequently, hydrogels emerge as a promising delivery platform, enhancing the efficacy of nucleic acid and viral drugs while minimizing adverse effects.

#### 3.4.1 Facilitate Efficient In Vivo Delivery In Vivo

The delivery of nucleic acid drugs typically relies on viral or nonviral vectors [268]. Viral vectors, while efficient, pose risks of immunogenicity and insertional mutagenesis [269]. Preexisting immunity or robust immune responses to viral vectors may compromise repeated dosing efficacy and safety. Lentiviruses, for instance, have been associated with insertional mutations that may contribute to oncogenesis [270]. Nonviral vectors, such as polyethylenimine (PEI)‐based nanoparticles, exhibit limited cellular uptake, with only ∼0.7% of administered doses reaching tumor tissue [271]. Upon entering the bloodstream, nanoparticles interact with plasma proteins (e.g., albumin, fibrinogen, apolipoproteins, complement proteins, and immunoglobulins), forming protein corona structures. These complexes are subsequently recognized by mononuclear phagocyte system receptors, leading to rapid clearance [272]. Hydrogels, however, provide a biocompatible and degradable alternative with superior safety profiles. Xu et al. [273] developed a branched‐chain PEI–GRAFT–PEG/PEO–PO–PEO/α‐CD hydrogel via host–guest interactions to directly encapsulate ABHD5 plasmid DNA for liver cancer immunotherapy. This approach bypasses traditional vectors, achieving transfection efficiencies of 73.7% in HEK293T cells, 43.6% in SMMC7721 cells, and 48.5% in SK‐Hep‐1 cells—significantly higher than the benchmark PEI‐25K vector. The hydrogel‐mediated delivery of ABHD5 plasmid to tumors reduced PD‐L1 expression, activated antitumor T‐cell responses, and inhibited tumor growth. Hydrogels excel in physical encapsulation and localized retention, ensuring plasmid stability and targeted delivery. While transfection efficiency and systemic targeting require further optimization, their inherent safety and multifunctional design make them ideal for tumor gene therapy. Plasmid DNA can be integrated into DNA hydrogels, preserving its biological activity while enabling programmable drug delivery. Wang et al. [104] developed a nucleic acid hydrogel (SCP‐NA‐Gel) by crosslinking Y‐shaped DNA motifs with PD‐L1 siRNA, achieving highly efficient coloading. The hydrogel enables spatiotemporally controlled release upon enzymatic degradation in tumors. 7‐ethyl‐10‐hydroxycamptothecin (SN38) induces ICD and antigen release, while CpG directly activates DCs, increasing their maturation rate to 42.6%. The siRNA downregulates PD‐L1 to relieve T‐cell suppression, collectively promoting deep infiltration of cytotoxic T lymphocytes, with CD8^+^ T cells reaching 36.6%. This approach significantly inhibited both primary and distant tumor growth in a melanoma model. Sequence programmability and biocompatibility further enhance their utility in precision gene therapy. In another study, Song et al. [274] encapsulated the G5‐BGG/shRNA871 plasmid complex within a PLGA–PEG–PLGA hydrogel to protect it from in vivo degradation, downregulate CD47 expression, and promote macrophage infiltration in residual tumor tissue. An in situ postsurgical tumor model demonstrated superior antiresidual tumor efficacy, with the hydrogel preserving nucleic acid stability and enabling localized, responsive release. Despite limitations in transfection efficiency and systemic targeting, the low immunogenicity and biocompatibility of hydrogels position them as a leading platform for nucleic acid delivery.

#### 3.4.2 Avoid Enzymatic Degradation

The phosphodiester bonds in native nucleic acids represent the primary target sites for nucleases [275]. Due to their structural properties, these bonds are inherently susceptible to enzymatic cleavage in vivo, leading to hydrolysis and subsequent loss of drug activity. Nuclease‐induced degradation of nucleic acid drugs can occur through either specific or nonspecific mechanisms, significantly reducing therapeutic efficacy. To address this challenge, researchers have focused on developing strategies to enhance the stability and bioavailability of nucleic acid therapeutics. In recent years, hydrogels have emerged as a promising delivery platform. Their unique three‐dimensional network architecture enables physical encapsulation of nucleic acid drugs, shielding them from enzymatic degradation while maintaining long‐term stability and improving bioavailability [244]. This approach offers a novel solution to the vulnerability of nucleic acid drugs to in vivo degradation.

In a study by Jia et al. [276], alginate hydrogels were utilized to encapsulate lipid nanoparticle (LNP)‐loaded ovalbumin (OVA)‐mRNA for melanoma immunotherapy. The researchers demonstrated that alginate gel effectively protected mRNA‐LNPs from environmental nucleases. Gel electrophoresis revealed that mOVA‐LNP bands disappeared after 3 days at room temperature, whereas hydrogel‐encapsulated mOVA‐LNP remained intact for 14 days and retained stability for 21 days without degradation. Local injection of the hydrogel‐encapsulated mOVA‐LNP significantly enhanced CD8^+^ T cell infiltration (1.65%) compared with direct intratumoral injection of mOVA‐LNP (0.48%) or free mOVA (0.29%). A major limitation of mRNA therapies is the rapid degradation caused by ubiquitous nucleases in biological and environmental settings. Hydrogels, with their water‐soluble three‐dimensional matrices, not only protect mRNA from enzymatic attack but also enable controlled, on‐demand release. This dual functionality mitigates mRNA hydrolysis while prolonging drug activity, thereby enhancing therapeutic outcomes. Yin et al. [232] further demonstrated the protective capacity of hydrogels by formulating a GO–polyethyleneimine hydrogel (GLP) through electrostatic interactions. The GLP hydrogel encapsulated OVA–mRNA (mOVA) for melanoma immunotherapy. While free RNA was degraded within 6 h in serum, GLP‐encapsulated mOVA retained detectable activity after 48 h of incubation. This protection against nuclease activity resulted in significantly elevated anti‐OVA IgG levels (∼600 ng/mL) in treated mice compared with direct mOVA injection, which showed negligible immune response. The enhanced immune activation effectively suppressed melanoma growth and metastasis.

For plasmid and RNA interference (RNAi) therapeutics, hydrogel encapsulation ensures sustained stability and function. Circular, supercoiled plasmid DNA is inherently stable but remains vulnerable to topoisomerase‐induced structural alterations and nuclease‐mediated cleavage in dynamic cellular environments [277, 278, 279]. Unprotected plasmid DNA is rapidly cleared from circulation. Zhou et al. [280] encapsulated a dual‐functional plasmid (pBMP‐2–VEGF, BEV–BP) and IL‐4 in GelMA hydrogels. IL‐4 modulates immune responses to reduce chronic inflammation, while BEV–BP synergistically promotes osteogenesis and angiogenesis through BMP‐2 and VEGF upregulation. The hydrogel platform temporally coordinates therapeutic stages, preserving plasmid activity and facilitating siRNA internalization. However, siRNA stability is compromised under low pH or elevated temperatures, as partial strand dissociation exposes single‐stranded regions to degradation. To overcome these limitations, Zhao et al. [281] developed a temperature‐sensitive PLEL hydrogel capable of delivering siRNA with high transfection efficiency. The hydrogel effectively inhibited siRNA degradation by nucleases, enhancing gene silencing efficacy. In comparative studies, the hydrogel group exhibited the strongest inhibition of HCC progression, underscoring its potential as a robust delivery system for RNA‐based therapies.

#### 3.4.3 Enhance Targeted Delivery Capabilities

A critical limitation of nucleic acid drugs in vivo lies in their suboptimal targeting capabilities [282]. These therapeutics must navigate complex physiological barriers and are prone to nonspecific cellular uptake and enzymatic degradation during delivery, hindering precise localization to target cells or tissues. This lack of specificity may result in unintended off‐target effects, potentially causing harmful systemic toxicity [283]. To address this challenge and improve the therapeutic index of nucleic acid drugs, researchers have focused on developing strategies to enhance drug–cell interaction specificity and cellular internalization efficiency. A promising approach involves leveraging hydrogels as delivery vehicles. Direct application of hydrogels at tumor sites enables localized, in situ delivery of nucleic acid therapeutics [284]. This strategy not only enhances spatial targeting accuracy but also minimizes exposure of nontarget cells to the drug, thereby reducing the risk of adverse effects.

The application of oncolytic viruses (OVs) in tumor immunotherapy represents a transformative approach [285]. However, off‐site viral dissemination remains a major obstacle. While direct intratumoral injection of OVs effectively targets neoplastic cells, preclinical studies have reported hepatotoxicity associated with viral migration from tumor sites to the liver [286]. This systemic distribution limits the therapeutic window of OVs and raises safety concerns. In a study by Du et al. [23], gelatin hydrogels loaded with OVs encoding IL‐12 and IL‐15 were employed for immunogene therapy in colon and lung cancer models. Quantitative analysis of viral genomic RNA in tumors and organs revealed that hydrogel‐encapsulated OVs exhibited significantly reduced hepatic accumulation (at 24 and 60 h postinjection) compared with free OVs. Conversely, tumor‐specific viral retention was enhanced, demonstrating the hydrogel's capacity to restrict off‐target viral distribution. Notably, mice treated with OV‐loaded hydrogels demonstrated a 5‐day extension in median survival compared with those receiving unencapsulated OVs.

As a next‐generation biomaterial, hydrogels occupy a pivotal role in modern drug delivery systems due to their exceptional biocompatibility and tunable physicochemical properties. Their ability to evade host immune recognition ensures safe in vivo utilization, while their structural adaptability permits site‐specific administration or implantation. This controlled spatial confinement enhances local drug concentrations at target sites while minimizing systemic exposure, a critical requirement for precision oncology and immunotherapy. Recent advancements in RNAi therapeutics have further underscored the importance of targeted delivery. siRNA demonstrates potent gene‐silencing capabilities but often exhibits poor in vivo targeting efficiency, leading to heterogeneous drug distribution and inconsistent therapeutic outcomes [287]. Frequent dosing requirements imposed by this limitation may compromise patient compliance and increase systemic toxicity risks. In a study by Lu et al. [288], alginate hydrogels were utilized to deliver B‐RAF siRNA nanoparticles for melanoma immunotherapy. Fluorescence imaging of B16F10 tumor‐bearing mice revealed that siRNA accumulation at tumor sites was fourfold higher in hydrogel‐treated groups compared with free siRNA nanoparticles after 120 h. This enhanced localization correlated with significantly suppressed MAPK signaling activity in tumors, as evidenced by a 15.3% activation of DCs and 14.6% infiltration of cytotoxic T cells. The sustained release profile of hydrogel‐encapsulated siRNAs not only amplifies local therapeutic efficacy but also reduces dosing frequency, thereby mitigating systemic side effects. These findings highlight the translational potential of hydrogel‐based systems in overcoming the inherent limitations of nucleic acid therapeutics. By enabling precise spatial control of drug distribution, hydrogels simultaneously enhance therapeutic potency and safety, offering a novel paradigm for the development of next‐generation biologics in oncology.

#### 3.4.4 Mitigating Virus‐Specific Immune Responses

Adenovirus is a large (36 kb) unenveloped double‐stranded DNA virus that enters cells through endocytosis [289]. Following internalization, its genome translocates to the nucleus, where it remains extrachromosomal and does not integrate into the host genome [290]. Despite its high transfection efficiency, adenovirus elicits robust innate and adaptive immune responses, which may compromise therapeutic efficacy and induce adverse effects [291]. To address this limitation, hydrogel‐based sustained‐release systems have been proposed as a strategy to modulate virus‐specific immune activation. By controlling viral release kinetics, reducing systemic exposure, and enhancing localized delivery, hydrogels can attenuate immune overactivation, thereby mitigating toxic side effects, improving therapeutic outcomes, and enhancing the safety profile of adenoviral vectors. Studies by Lv et al. [292] provide experimental validation of this approach. They encapsulated oncolytic adenovirus (OV) within an alginate hydrogel for the immunotherapy of HCC. Results demonstrated that the antiviral immune response elicited by unencapsulated OV was significantly higher than that observed with OV hydrogel formulations. Conversely, the antitumor immune response was more pronounced in the hydrogel group, indicating that hydrogel encapsulation reduces viral immunogenicity while potentiating antitumor activity. Notably, intratumoral infiltration of CD4^+^ and CD8^+^ T cells in OV‐treated tissues was quantified at 7% and 12.1%, respectively, compared with control tissues. In contrast, CD68^+^ macrophage infiltration levels were 6.18 and 5.20% in the respective groups (a correction to the original text, as CD68^+^ cells are macrophages, not CD8^+^ T cells).

Adenovirus‐induced immune activation, while therapeutically beneficial in some contexts, poses challenges due to its potential to trigger systemic toxicity. Hydrogels effectively mitigate immune‐related adverse effects by acting as a physical barrier to neutralizing antibodies and inflammatory mediators. This dual functionality ensuring targeted delivery to tumor sites while protecting the virus from rapid clearance, enhancing the virus's persistence and oncolytic efficacy. For instance, Zheng et al. [293] employed a PLGA–PEG–PLGA hydrogel to deliver Sendai virus, demonstrating a significant reduction in acute inflammatory responses compared with conventional subcutaneous injection. Localized direct injection of viruses, while advantageous for targeting lesion sites, risks premature immune‐mediated clearance and systemic adverse reactions. Hydrogels address this limitation by creating a protective microenvironment that shields the virus from host immune surveillance, thereby prolonging its half‐life and therapeutic window. The study by Wu et al. [294] further demonstrated the protective function of hydrogels. They developed a silk fibroin hydrogel‐supported PD‐L1‐targeting CRISPR–Cas9 adenoviral vector (SgCas9–AdV). In this study, mice administered with the SgCas9–AdV hydrogel showed a serum adenovirus neutralizing antibody level of approximately 0.2 OD over 9 consecutive days, compared with 0.6 OD in mice receiving unencapsulated SgCas9–AdV via direct intratumoral injection. Furthermore, the hydrogel group exhibited enhanced T‐cell infiltration, with CD4^+^ T cells increasing to 40.4% (vs. 19.0% in the control) and CD8^+^ T cells reaching 23.9% (vs. 23.0% in the control), highlighting the hydrogel's ability to enhance immune‐mediated tumor targeting. These findings collectively underscore the translational potential of hydrogel‐based systems in overcoming immune barriers associated with adenoviral therapy. By coordinating controlled viral release with immune evasion mechanisms, hydrogels offer a promising platform to improve the safety and efficacy of viral vectors in cancer immunotherapy.

### 3.5 Hydrogel Containing Cell

In recent years, cell therapy has emerged as a prominent research domain within the broader field of tumor biotherapy, demonstrating considerable promise in cancer treatment. This approach involves the infusion of active immune cells, such as T cells, NK cells, and DCs, to enhance the immune function of tumor patients, with the goal of suppressing or eradicating tumors [295, 296]. The abundance of tumor antigens within neoplastic cells provides a diverse array of targets for the immune system, thereby facilitating the development of personalized immunotherapeutic strategies [297]. The integration of cell therapy with hydrogel technology holds significant potential, further expanding its therapeutic applications. Hydrogels have demonstrated the ability to facilitate targeted delivery of drugs to the target site, enabling precise localized therapy [298]. Specifically, immune cells can be encapsulated within specialized hydrogels that not only preserve their functionality but also ensure their stability and viability in vivo [10]. Furthermore, embedding immune cells within hydrogel matrices enhances their implantability, streamlining the treatment process and improving clinical efficacy [299]. Importantly, hydrogels fulfill the requirement for sustained release of cellular components; by modulating the release kinetics, an effective and prolonged immune response can be maintained, resulting in extended systemic immune activation [176, 300]. This characteristic is particularly advantageous for diseases requiring long‐term monitoring and intervention, offering a novel therapeutic strategy that combines biomaterials with biotherapeutic approaches to deliver more customized and efficient treatments.

#### 3.5.1 Simulating Immune Microenvironments

The therapeutic efficacy of immune cells depends on their ability to remain viable and functionally active after administration [301]. Hydrogels provide a favorable microenvironment that supports immune cell survival and activity [302]. Their tunable mechanical properties, such as flexibility and elasticity, closely mimic the physical characteristics of native tissues, which is critical for maintaining immune cell morphology and function [303]. Additionally, the porous architecture of hydrogels allows for the free diffusion of oxygen, nutrients, and metabolic waste products. This ensures adequate nutrient supply and oxygenation while enabling the removal of waste metabolites, preventing toxic accumulation that could compromise immune cell viability [304, 305]. Consequently, hydrogels offer a biocompatible platform conducive to immune cell activity and long‐term functionality. In this context, hydrogels serve as promising carriers or scaffolds in cell‐based therapies, significantly enhancing therapeutic outcomes and opening new avenues for immunotherapy development.

Jie et al. [306] developed a multifunctional hydrogel scaffold based on self‐assembling peptides that mimics the physicochemical properties of the TME, providing a biomimetic 3D proliferation platform for CAR‐T cells. Hydrogel encapsulation significantly extended the retention time of CAR‐T cells within the tumor for over 7 days and promoted their sustained activation and expansion, achieving a more than 12‐fold increase in cell numbers within just 3 days. In vivo results demonstrated that tumors treated with hydrogel‐loaded CAR‐T cells showed a significantly higher density of infiltrating CAR‐T cells, with the proportion of effector T cells (CD8^+^ IFN‐γ^+^) increasing approximately threefold. This was accompanied by markedly elevated secretion levels of interferon‑γ and granzyme B. By integrating mechanotransduction signals and localized immunomodulation, this biomimetic scaffold effectively maintains T cell functional activity, offering a novel technological platform for local immunotherapy of solid tumors.

Traditional adoptive cell transfer (ACT) protocols typically require 2–4 weeks to generate functional CAR‐T cells, during which time the cytotoxic and memory capabilities of these cells may decline [307, 308]. To address this limitation, Santos et al. [309] employed a porous PEG–heparin hydrogel to codeliver T cells and activating agents (dynabodies), promoting efficient T cell proliferation. The hydrogel exhibited a uniform pore size of approximately 80 µm, allowing unrestricted migration and expansion of T cells, which typically measure around 7 µm in diameter. The storage modulus of the hydrogel closely resembled that of lymphoid tissues. Compared with conventional activation methods, the hydrogel‐based approach showed superior preservation of T cell viability (26.1% PI^+^) and enhanced proliferation rates (a 1.63‐fold increase over traditional methods). Thus, the hydrogel effectively recapitulated key aspects of lymphoid tissue architecture, simulating the activation and expansion processes of T cells in vitro. Relative to standard methodologies, this system has the potential to significantly reduce both the time and cost associated with ACT preparation.

The three‐dimensional network structure of hydrogels closely resembles that of the natural extracellular matrix, providing essential mechanical support and attachment points for embedded cells [310]. Beyond merely replicating the structural features of native tissues, hydrogels actively support cellular functions such as adhesion, proliferation, and differentiation [311]. These attributes position hydrogels as ideal candidates for use as cell carrier materials in various biomedical applications. The advancement of cell therapy has been significantly propelled by hydrogel technologies, which have also offered innovative solutions to persistent challenges in current medical practice [312]. Rapid intracellular hydrogelation of DCs using PEGDA hydrogel monomers have enabled the formation of robust and modular cell‐hydrogel hybrids, with minimal disruption to cell morphology or antigen presentation capabilities. In a related investigation, Yang et al. [313] utilized 3D printing technology to prepare a gelatin–alginate saline gel based on living immune cells of macrophages (M_0_‐RAW264.7). Hydrogels can not only provide favorable survival conditions for macrophages, but also generate a large amount of ROS, which subsequently polarize macrophages into the M1 phenotype with antitumor ability. By secreting cytotoxic cytokines, it effectively induces apoptosis of tumor cells.

#### 3.5.2 Promote Local Colonization

In the field of tumor immunotherapy, immune cells and tumor cells play a central role [314]. Various mechanisms have been explored to enhance the host's ability to recognize and eliminate tumor cells. However, successful cell therapy typically requires effective engraftment at the target site and sustained stimulation of the immune system [315, 316]. Hydrogels exhibit favorable biocompatibility, chemical modifiability, and physical adaptability. Certain types of hydrogels remain in a liquid state at room temperature and undergo rapid gelation after injection into the body—features that make them particularly suitable for minimally invasive procedures or direct delivery to the TME [116, 317]. These characteristics enable hydrogels to serve as carriers for implantable cellular components, facilitating their localization and functional deployment at the tumor site.

The immunosuppressive nature of the solid TME has been shown to impair the antitumor activity of CAR‐T cells [318]. Hu et al. [319] developed an implantable hyaluronic acid‐based hydrogel capable of integrating CAR‐T cells directly into the tumor bed following surgical resection of subcutaneous melanoma in mice. This approach effectively inhibited both local recurrence and distant tumor growth. The hydrogel demonstrated a high loading efficiency of 97.3% for CAR‐T cells. In vivo imaging system (IVIS) analysis revealed that the hydrogel remained localized at the tumor site for approximately 8 weeks. Experimental results further indicated that the hydrogel group exhibited superior efficacy in controlling local tumor recurrence and inducing systemic antitumor immunity. Currently, CAR‐T cell therapy is predominantly administered via intravenous infusion. This route allows for easy monitoring of T cells in the bloodstream and facilitates their interaction with circulating tumor cells, thereby demonstrating significant efficacy in hematologic malignancies. However, this systemic delivery approach presents notable limitations in the context of solid tumors. Specifically, when delivered intravenously, CAR‐T cells often encounter difficulties in infiltrating solid tumors due to the complex architecture and dense extracellular matrix characteristic of these lesions. Moreover, the immunosuppressive TME further compromises CAR‐T cell function and expansion [320, 321].

Despite the clinical success of CAR‐T cell therapy in treating blood cancers, major challenges remain in its application to solid tumors [322]. To overcome these obstacles, researchers are investigating various strategies, including the integration of hydrogels and other biomaterials as advanced delivery platforms. These approaches aim to improve the localization, survival, and therapeutic efficacy of CAR‐T cells within the TME. In a recent study, Zhou et al. [323] developed a photocurable GelMA hydrogel that enables controlled release of CAR‐T cells around the tumor site, with sustained release lasting over 5 days, significantly enhancing local cell colonization and infiltration. By modulating the hydrogel degradation rate, the system controls the spatiotemporal distribution of T cells, increasing the proportion of central memory T cells within tumors and enhancing the expression of activation markers CD69 and CD25. In vivo results showed that the hydrogel treatment group achieved complete disappearance of bioluminescent tumor signals by Day 26, along with a reduced proportion of TIM‐3^+^ T cells in tumors and a median survival extended beyond 60 days, significantly outperforming the intravenous injection group. This local delivery system enhances antitumor immune responses by maintaining T cell activity and memory phenotypes. T cell‐based therapies have demonstrated promising clinical outcomes; however, they require extensive and costly in vitro manufacturing processes, which can result in heterogeneous cell products [324, 325]. Although CAR‐T cell therapy has achieved considerable clinical success and provided new hope for many patients, the production process remains associated with significant logistical and technical challenges. The generation of CAR‐T cells involves multiple labor‐intensive steps, leading to high production costs and potential variability across batches. This heterogeneity may compromise the consistency and reproducibility of therapeutic effects, necessitating additional quality control measures for clinical applications [326, 327]. Consequently, current research efforts are focused on developing strategies to streamline the manufacturing pipeline, reduce costs, and improve the uniformity of final cell products. For instance, Agarwalla et al. [183] introduced an implantable, multifunctional alginate‐based T cell engineering and release scaffold (MASTER), designed to simplify in vivo CAR‐T cell manufacturing. The scaffold enables uniform distribution of T cells within its porous structure, promoting their engraftment at the tumor site. Compared with traditional intravenous CAR‐T cell infusion, the MASTER platform demonstrated superior tumor control and significantly prolonged survival in preclinical models.

#### 3.5.3 Act as an Adjuvant

A widely adopted form of tumor cell vaccines employs inactivated or genetically modified autologous tumor cells, as well as tumor cell lysates, as antigen sources [328]. The therapeutic protocol involves isolating tumor cells from the patient and subjecting them to physicochemical or biological treatments that render the cells nontumorigenic while preserving their tumor‐specific antigens capable of eliciting an immune response [329]. This approach not only minimizes the risk of adverse effects during therapy but also ensures a highly specific immune reaction. Upon readministration into the patient, these treated tumor cells are captured by APCs, such as DCs. DCs subsequently process and present the tumor‐derived antigens to T lymphocytes, thereby initiating a targeted immune response. However, the immunostimulatory potential of conventional tumor cell vaccines remains limited [330]. To address this limitation, researchers are actively investigating strategies aimed at enhancing both the speed and magnitude of the immune response, with the goal of improving therapeutic outcomes. Hydrogels have emerged as promising tools for augmenting the immunostimulatory capacity of tumor cell vaccines through multiple mechanisms, including the codelivery of adjuvants or immunomodulatory molecules, prolonged immune stimulation, and enhanced activation of APCs [195, 331, 332]. He et al. [333] developed a hydrogel system composed of crosslinked mannose and PEI to encapsulate mitoxantrone hydrochloride‐treated tumor cells for melanoma immunotherapy. Compared with direct treatment of RAW264.7 macrophages with untreated tumor cells, the hydrogel‐loaded formulation significantly increased the expression levels of CD40 and CD80 on RAW264.7 cells. Moreover, the proportion of CD8^+^ IFNγ^+^ T cells following treatment with the tumor cell‐hydrogel formulation reached 28.73%, surpassing the 21.57% observed in mice receiving direct injection of chemotherapy‐treated tumor cells. These experimental results demonstrate that hydrogels can effectively enhance the immunostimulatory properties of tumor cell vaccines.

As a personalized immunotherapeutic strategy, tumor cell vaccines aim to activate the patient's immune system to specifically recognize and eliminate tumor cells [334]. This approach is tailored to each individual based on the unique antigenic profile of their tumor, using components derived from the patient's own tumor tissue to prime the immune system for a targeted response [335]. However, to counteract the various immune evasion mechanisms employed by tumor cells, it is crucial to amplify the immunostimulatory effects of these vaccines [336]. Enhancing immune activation not only promotes a stronger and more durable response but also represents a key strategy for improving therapeutic efficacy [337]. In this context, hydrogels serve as advanced drug delivery platforms that offer novel perspectives and solutions for the development of tumor cell vaccines. Their distinctive structural features, such as a three‐dimensional network architecture and excellent biocompatibility make them ideal candidates for encapsulating and delivering tumor‐associated antigens, adjuvants, and even live cells. These components can be gradually released in vivo, thereby sustaining immune activation over time [338, 339]. Additionally, the degradation rate and pore structure of hydrogels can be precisely modulated to meet specific therapeutic requirements, ensuring optimal distribution and retention of vaccine components at the target site [340]. Yang et al. [133] investigated a CD–PEG‐based hydrogel loaded with DOX‐treated tumor cells for melanoma immunotherapy. The hydrogel formulation containing chemotherapeutically treated tumor cells exhibited a significantly higher percentage of DCs (25.6%) compared with the empty hydrogel control (1.56%). Furthermore, when used as a coincubation platform for DCs and tumor cells, the hydrogel formulation stimulated greater proliferation of CD8^+^ T cells (24.9%) and CD8^+^IFN‐γ^+^ T cells (21.1%) in the spleen than the direct mixing of tumor cells and DCs (11.4 and 15.4%, respectively). These findings indicate that hydrogel‐based tumor cell vaccines effectively promote DC recruitment and provide a functional platform for DC‐tumor cell interactions, facilitating efficient antigen processing and presentation.

Moreover, the application of hydrogels has been shown to substantially enhance the immunostimulatory effects of tumor cell vaccines, while simultaneously improving the safety and controllability of the treatment. For instance, the design of stimuli‐responsive hydrogels enables precise modulation of vaccine release in response to physiological signals such as pH variations [341, 342]. Smart hydrogel systems that respond to temperature changes or enzymatic activity allow for controlled delivery of vaccine components at specific times and locations [343]. Such advancements hold promise for optimizing immune activation while minimizing off‐target effects. This dual benefit not only improves vaccine efficacy but also reduces the likelihood of adverse events, paving the way for innovative approaches in tumor immunotherapy. Therefore, the integration of hydrogel technology into tumor cell vaccine development is anticipated to drive significant breakthroughs in cancer treatment and advance the precision and effectiveness of personalized medicine [344]. In a separate study, Yoshizaki et al. [345] utilized a PCG‐based hydrogel system to codeliver DCs, OVA antigens, and CpG–DNA. Compared with the direct addition of CpG–DNA and OVA antigens to a DC cell suspension, the use of the hydrogel as a combined delivery platform resulted in a 1.5‐fold increase in TNF‐α expression in DCs. Furthermore, tumor size was significantly reduced, and median survival time was extended by approximately 3 days in animals treated with hydrogel‐codelivered DCs compared with those receiving isolated DCs after OVA and CpG–DNA treatment.

### 3.6 Hydrogel Containing Bacteria

Bacteria are a major group of living organisms. While bacteria are implicated in numerous diseases, they also play essential roles in various physiological processes. Notably, they contribute significantly to fermentation, generating beneficial outcomes such as the production of prebiotics [346]. Upon invasion into the human body, bacteria interact with the host microbiome and modulate the immune system, triggering adaptive immune responses [347, 348]. Consequently, bacterial immune regulation represents a promising approach to enhance the efficacy of cancer therapy.

In recent years, an increasing number of strategies have been developed for tumor treatment using bacteria. Bacteria‐mediated therapy has emerged as a versatile and evolving antitumor strategy [349]. In particular, hydrogels have gained attention as a promising delivery platform within the body. The porous structure of hydrogels enables the exchange of nutrients and metabolites, thereby maintaining microbial viability [350]. However, the application of bacterial therapy in oncology remains limited due to safety concerns, including high toxicity and lack of specificity. By contrast, the integration of hydrogels enhances safety through physical containment and controlled release mechanisms [351].

#### 3.6.1 Promote Bacteria Growth

Hydrogel, a material characterized by a three‐dimensional network structure, can provide a tailored and controllable microenvironment conducive to bacterial growth [303]. The physicochemical properties of hydrogels, including porosity, water content, pH, charge characteristics, and surface chemistry—significantly influence bacterial adhesion and proliferation [352]. For instance, optimal porosity facilitates the efficient diffusion of oxygen and nutrients, while adequate water content sustains a moist environment favorable for microbial survival [353]. Moreover, strategic modulation of the hydrogel's pH and surface charge can optimize interactions with specific bacterial species, thereby promoting adhesion and subsequent growth. With precise engineering, hydrogels can be designed to release essential nutrients that support bacterial proliferation, further enhancing microbial activity [354].

These functional hydrogels find applications in scientific research, where they can simulate complex biofilm formation, as well as in the development of novel probiotic delivery systems. For example, in the context of gut microbiome studies or agricultural microbiology, this property can be harnessed to increase the abundance and activity of beneficial microorganisms, aiming to improve host health or boost crop productivity [355, 356]. Thus, advances in hydrogel technology hold considerable promise for both fundamental research and practical applications in microbiology. Pan et al. [357] developed a self‐adjuvanting bacterial hydrogel system (AraGel@ARB), which utilizes an arabinose‐based hydrogel to provide a supportive microenvironment for engineered bacteria, sustaining their viability and regulating their immunomodulatory functions. The spatiotemporally controlled release from the hydrogel enhances the maturation of DCs in tumor‐draining lymph nodes, promotes the infiltration of CD4^+^ and CD8^+^ T cells (increased by approximately fivefold and eightfold, respectively), and significantly upregulates key cytokines including TNF‐α, IL‐6, IL‐12p70, and IFN‐γ, strongly activating the TLR pathway. In vivo studies confirm that this system effectively activates antitumor immunity without inducing significant tissue inflammation. Hydrogels can be precisely formulated to promote the survival and proliferation of beneficial bacterial strains while inhibiting the growth of potentially pathogenic species by adjusting the chemical composition, structural features, and release kinetics [358]. This capacity to finely regulate the internal microenvironment is particularly valuable in developing personalized therapies tailored to individual microbiome profiles. In a notable study, Zheng et al. [359] developed a mucoadhesive hydrogel loaded with silver nanoparticles (Agel), which modulates the oral microbiome by selectively inhibiting competing bacteria while promoting the survival of *Peptostreptococcus*. Agel enables localized and sustained release in the oral cavity, enhancing anaerobic bacteria‐induced DC maturation (significantly increased CD80^+^CD86^+^ cell proportion) and promoting CD8^+^ T cell infiltration in both SCC7 and 4MOSC1 mouse models, thereby synergistically enhancing antitumor immune responses. This system showcases the potential of hydrogels as a microbial immunomodulatory platform with spatiotemporally controlled release. Hydrogels have demonstrated a wide range of potential applications in the biomedical field due to their excellent biocompatibility. These materials not only support cellular growth but also mimic the natural extracellular matrix, helping maintain cell viability and functionality. Additionally, they serve as protective barriers, shielding bacteria from environmental stressors such as oxidative damage, temperature fluctuations, and exposure to harmful chemicals. Qiu et al. [360] developed an injectable chondroitin sulfate hydrogel capable of supporting sulfate‐reducing bacteria (SRB). The hydrogel not only sustained SRB growth but also supplied sulfate to enable prolonged hydrogen sulfide production over at least 7 days. This process induced mitochondrial dysfunction and ICD, thereby augmenting antitumor immunity.

#### 3.6.2 Improve Biocompatibility

The diverse array of bacteria, coupled with their widespread availability, has led to innovative concepts and methodologies for tumor treatment [361]. However, the application of these approaches is constrained by the inherent unpredictability of bacterial behavior and potential safety concerns [362]. These organisms may proliferate uncontrollably within the body, disseminating to nontarget areas, thereby compromising therapeutic efficacy and potentially inducing severe adverse effects. To address these challenges, researchers are developing various strategies to enhance the safety and controllability of bacterial therapies [363, 364]. Among these, hydrogels have emerged as a promising solution for drug delivery. By encapsulating bacteria within a hydrogel matrix, this approach establishes a physical barrier that prevents bacterial escape to nontargeted regions, reduces impact on normal tissue, and decreases the likelihood of systemic exposure, significantly mitigating the risk of systemic side effects [365]. Additionally, hydrogels can modulate the immune system's response to bacteria, ensuring biosafety while maintaining therapeutic efficacy. Meng et al. [366] prepared a Pluronic F127‐based thermosensitive hydrogel for loading Microcystis aeruginosa (MA). This strategy enhances the precision and safety of bacterial therapies and provides a novel method for developing tumor treatments. Injecting a hydrogel precursor solution into tumor tissue allows immediate conversion to hydrogel, confining MA to tumor tissue and preventing its toxic effects on other tissues. MA upregulates ICD markers CRT (control: 0.21, MA: 37.14) and HMGB1 (control: 0.213, MA: 2.337). In vivo imaging demonstrated that the hydrogel could remain accurately within the tumor for 48 h without leakage into other major organs. The utilization of bacteria in tumor immunotherapy represents an emerging area of research. Through the employment or modification of specific bacterial species, scientists have explored numerous methodologies to stimulate the host's immune system, thereby facilitating cancer combat. To ensure safety and controllability, hydrogels emerge as an optimal medium for drug delivery. Compared with their active, living counterparts, spores exhibit a state of dormancy, conferring enhanced safety and stability. Nevertheless, the development of suitable delivery carriers remains a critical step in achieving in vivo delivery and ensuring safety. Hydrogels exhibit favorable biocompatibility and can be engineered to release spores in response to specific environmental cues, such as pH, temperature, or enzyme presence. This approach enhances the safety and efficacy of the treatment, maximizing the unique advantages of spores, providing patients with a more accurate and safe approach to tumor immunotherapy. Zhu et al. [367] encapsulated C. novyi‐NT spores with a melittin‐RADA32 hydrogel (MR) to target hypoxic regions in glioblastoma. The hydrogel significantly enhanced the biocompatibility of the bacteria, as shown by HE and Gram staining, which confirmed no bacterial colonization in vital organs such as the heart, liver, spleen, or lungs, and no impact on blood glucose levels. In terms of immune regulation, the hydrogel, through its spatiotemporally controlled drug release, promoted DC maturation and antigen presentation, thereby enhancing CD8^+^ T cell infiltration and activation and induced M1 macrophage polarization, effectively remodeling the tumor immune microenvironment. As research progresses, the antitumor effect of bacteria has been increasingly substantiated and acknowledged, demonstrating its promise in tumor immunotherapy. Utilizing bacteria's inherent characteristics, such as their ability to thrive in low‐oxygen conditions, enables precise targeting of tumor sites. In this context, hydrogels have emerged as a promising delivery carrier, safeguarding bacteria from the complex environment in the body and ensuring their safe arrival at the tumor site. They also regulate the release rate of bacteria by adjusting the properties of the hydrogel, enhancing therapeutic effects and reducing side effects. In their study, Chen et al. [368] developed a delivery system based on the thermosensitive hydrogel P407. The sustained‐release properties and biocompatibility of hydrogels ensure that the immune response caused by engineered lactic acid (FOLactis) is not overly intense, effectively controlling the body's immune response to bacteria. In a separate study, Kremenovic et al. [369] developed a heat‐sensitive PLGA–PEG–PLGA hydrogel for delivering Mycobacterium bovis BCG lysate. When injected near tumors, this hydrogel exhibited reduced invasiveness, facilitating controlled release of therapeutic agents.

### 3.7 Application Principles in Tumor Immunotherapy

Hydrogels demonstrate significant potential across multiple application scenarios in tumor immunotherapy, enabling the customization of immune strategies according to specific requirements. More importantly, by integrating multiple functions into a single platform, hydrogels can exert synergistic effects, establishing a solid foundation for constructing a systematic combination therapy framework.

The application of hydrogels in tumor immunotherapy follows a precision‐matching principle, with tailored design of both therapeutic agent loading and material properties based on distinct treatment goals and immune microenvironments. In scenarios requiring enhanced immune stimulation, such as oral vaccines or postsurgical immunotherapy, hydrogels are suitable for loading protein antigens, molecular adjuvants, or tumor lysates. They must exhibit pH‐responsive swelling, mucoadhesive properties, and sustained‐release characteristics to protect antigen integrity and promote local immune activation [370, 371]. In contexts where reversal of immunosuppression is needed, such as combating T cell exhaustion, hydrogels can be loaded with immune checkpoint inhibitory peptides or small‐molecule agonists. The key design consideration here is achieving systemic delivery and sustained release of drugs to block inhibitory signals and remodel the TME [372, 373]. In the field of photothermal and photodynamic therapy (PDT), hydrogels serve as cocarriers for energy‐converting agents and immunotherapeutic drugs. For instance, near‐infrared‐I (808 nm)‐responsive hydrogels can simultaneously deliver photothermal agents and immune adjuvants. While PTT induces ICD in tumors, these hydrogels release adjuvants to activate DCs [374]. In contrast, near‐infrared‐II (1064 nm)‐responsive systems enable deeper tissue penetration and work synergistically with PDT to generate ROS, thereby promoting antigen release and T‐cell activation [375]. Additionally, some studies have coencapsulated visible light (660 nm)‐activated PDT photosensitizers and STING agonists within hydrogels [376]. This approach leverages the synergy between localized oxidative stress and innate immune activation to suppress the growth of distal tumors. Furthermore, in scenarios requiring local and sustained release of cytokines or small‐molecule drugs, hydrogel design emphasizes high loading capacity and controlled release kinetics to maintain effective local concentrations while avoiding systemic toxicity [377, 378]. In applications specifically targeting the TME, key factors influencing immunotherapy efficacy include hypoxia, acidic pH, elevated levels of ROS, and high concentrations of GSH and specific enzymes [180, 379, 380]. Hydrogels must be capable of sensing and responding to these unique microenvironmental signals to achieve precise, on‐demand drug release. Hydrogels can be loaded with small‐molecule drugs that directly interfere with immunosuppressive cells or reverse inhibitory signals and creating a long‐term, local drug reservoir [9, 381]. Through the sustained release of these immunomodulatory agents, hydrogels can continuously neutralize immunosuppressive factors in the peritumoral area or postsurgical cavity, promoting the establishment of a proinflammatory microenvironment. This transforms “cold” tumors into “hot” tumors, creating favorable conditions for subsequent T cell infiltration and attack [382]. For combination therapy scenarios, where monotherapy efficacy is limited or coordination of multiple immune mechanisms is required, hydrogels serve as an ideal codelivery platform to integrate various treatment modalities and achieve synergistic effects. Examples include coloading antigens and adjuvants to enhance DC activation, or codelivering chemotherapeutic agents and immune agonists to synergize ICD with innate immune activation [383, 384, 385]. The design of such systems must focus on temporal control and spatial targeting of drug release to maximize synergy and minimize systemic toxicity.

In summary, through precise functional design, hydrogels can be adapted to diverse scenarios in tumor immunotherapy, providing a powerful technological platform for achieving efficient and safe precision immunotherapy.

## 4 Clinical Progress

Hydrogel is a hydrophilic polymer material with a three‐dimensional network structure. It has attracted much attention due to its excellent biocompatibility, adjustable physical and chemical properties, and intelligent response to external stimuli. These attributes make it widely applicable in various medical scenarios, demonstrating broad prospects in the field of oncology. This article briefly reviews the current status of hydrogel systems that are either commercially available or in clinical research, with an aim to integrate advanced functionalities and improved applications into cancer immunotherapy. A summary of the clinical progress is provided in Table 3.

**TABLE 3:** Clinical trial progress.

Drug name (company)	Hydrogel materials/payloads	Route of administration	Mechanism	Approval indication	Approval (year)	Clinical trials number	Study phase
TC‐3 Gel (UroGen Pharma)	Reverse thermal gel/mitomycin C (MMC)	Intravesical instillation	Liquid at low temperature, forms gel at body temperature, sustained release of MMC	NMIBC	Clinical development terminated	NCT01799499	Status: withdrawn
UGN‐102 (UroGen Pharma)	Reverse thermal gel/mitomycin C (MMC)	Intravesical instillation	Liquid at low temperature, forms gel depot at body temperature, sustained drug release	LG‐IR‐NMIBC	2024 (FDA)	NCT04688931	Marketed
UGN‐103 (UroGen Pharma)	Reverse thermal gel/mitomycin C (MMC)	Intravesical instillation	Liquid at low temperature, forms gel depot at body temperature, sustained drug release	LG‐IR‐NMIBC	Clinical research phase	NCT06331299	Phase III
LICoRN‐01	Hydrogel/GM‐CSF; mifamurtide	Intraoperative implantation into ablation cavity	Local sustained release of immunomodulators, activates antitumor immunity	Colorectal cancer liver metastases	Clinical research phase	NCT04062721	Phase I
STM‐416p	Biodegradable hydrogel	Intraoperative implantation into resection cavity	Local long‐term retention and sustained drug release	Prostate cancer (postoperative)	Clinical research phase	NCT06450106	Phase I
STM‐416	Biodegradable hydrogel	Implantation into resection cavity	Local long‐term retention and sustained drug release	Recurrent bladder cancer	Clinical research phase	NCT05710848	Phase I/IIa
HepaSphere	Superabsorbent polymer hydrogel/irinotecan, doxorubicin	Transarterial chemoembolization (TACE)	Occludes tumor vasculature and loads/sustains release of chemotherapeutic drugs	HCC, colorectal cancer liver metastases	Marketed (FDA 2006; NMPA 2014)	NCT04866290; NCT01387932	Marketed
DC Bead‐LUR	Microsphere carrier/lurbinectedin	Transarterial chemoembolization (TACE)	Loads chemotherapeutic drug, slow release in tumor vasculature	Colorectal cancer liver metastases	Clinical research phase	NCT02525380	Phase II (previous status)

The clinical trials data sources: https://clinicaltrials.gov/.

Currently, locally perfused hydrogels based on thermoreversible properties have emerged as a promising strategy in experimental clinical treatments for tumors [386]. A core feature of such hydrogels is their “reverse thermal phase transition” capability: they remain in a flowable liquid state at room temperature, facilitating injection or perfusion, and rapidly transition into a semi‐solid gel state at body temperature to forming a local drug reservoir at the lesion site [387]. TC‐3 hydrogel is one such material with “reverse thermal characteristics.” Prior to injection, TC‐3 hydrogel is mixed with the chemotherapeutic drug mitomycin C (MMC) at low temperatures, remaining liquid for easy intravesical instillation via standard catheters. Upon entry into the bladder, TC‐3 hydrogel rapidly transforms into a gel at body temperature and forming a drug reservoir. This gel reservoir adheres to the bladder wall and gradually dissolves over time, enabling sustained release of MMC. It is used in the treatment of nonmuscle‐invasive bladder cancer (NMIBC) and muscle‐invasive bladder cancer, addressing limitations of traditional intravesical therapy such as rapid drug clearance due to urine dilution and frequent voiding [388, 389]. In 2013, UroGen Pharma initiated a prospective, randomized, open‐label, dose‐ranging comparative study to explore the clinical feasibility of this formulation (NCT01799499). Although subsequent clinical development of this specific formulation appears to have been discontinued, the company continued research and development within its reverse thermal gel technology platform and successfully launched UGN‐102 (Zudifrir). UGN‐102 is also a MMC‐based gel formulation for the treatment of NMIBC. In the Phase III ENVISION trial, it demonstrated positive results, with a complete response rate of 79.6% at 3 months posttreatment and durable efficacy (NCT04688931) [390, 391]. Supported by the results of the Phase III ENVISION study, UGN‐102 received United States Food and Drug Administration (US FDA) approval on June 12, 2024, becoming the first US FDA‐approved drug for the treatment of low‐grade intermediate‐risk NMIBC [392]. UGN‐103, an improved or optimized version of UGN‐102, is currently still in clinical development. A Phase III single‐arm study named UTOPIA is ongoing to evaluate its efficacy and safety, planning to enroll approximately 92 patients (NCT06331299). Its goal is to provide a potentially superior treatment option for this disease [393].

The high water content of hydrogels helps maintain drug activity, their sustained‐release mechanisms prolong the duration of action, and their biocompatibility reduces the risk of immune rejection. Intraoperatively implanted hydrogels for immunomodulation, applied during tumor resection surgery as local sustained‐release carriers for immunomodulators, hold promise for activating antitumor immune responses to prevent postoperative recurrence [394]. Several related formulations have entered clinical trials. LICoRN‐01 is a local immunotherapy for colorectal cancer liver metastases. It serves as a carrier loaded with two immunomodulators: GM‐CSF (granulocyte‐macrophage colony‐stimulating factor) and mifamurtide (a TLR agonist). This formulation is injected in situ into the ablation zone after the tumor undergoes radiofrequency ablation. The hydrogel forms a local drug reservoir that enables sustained release of the immunotherapeutic agents, thereby recruiting and activating immune cells such as DCs, aiming to trigger a potent and durable antitumor immune response to eliminate residual lesions and reduce the risk of recurrence [395]. Research on LICoRN‐01 is currently in Phase I clinical trials, primarily aiming to assess the safety and feasibility of this treatment approach (NCT04062721). STM‐416p is another intraoperative immunotherapy based on biodegradable hydrogels. It is designed as a hydrogel formulation that can be directly implanted into the surgical cavity after tumor removal during cancer surgery. This hydrogel can reside locally for an extended period and slowly release its carried drug. It is currently under investigation in a Phase 1 clinical trial for prostate cancer patients undergoing radical prostatectomy (NCT06450106). STM‐416, a candidate therapy utilizing the same technology platform as STM‐416p, is also being evaluated in another Phase 1/2a clinical trial for recurrent bladder cancer (NCT04866290) [396, 397].

Drug‐eluting microspheres are essentially micron‐sized hydrogel spheres that serve as core tools in interventional radiology for transarterial chemoembolization (TACE) [398, 399]. Several microsphere‐related formulations are either in clinical trials or have been commercially approved. HepaSphere microspheres are superabsorbent polymer hydrogels. They are small in their dry state but can rapidly swell to several times their original size upon fluid absorption. This property allows them to better occlude blood vessels and concentrate drugs within the tumor site. Their three‐dimensional hydrogel network structure can load chemotherapeutic drugs and provide sustained, slow release locally within the tumor, maintaining high intratumoral drug concentrations over time [400, 401, 402]. HepaSphere microspheres received approval from the US FDA in 2006 and from China's CFDA/NMPA in 2014, and have been used clinically [403]. Concurrently, clinical research continues to advance. For instance, a prospective registry study on chemoembolization using irinotecan‐loaded HepaSphere microspheres in patients with colorectal liver metastases (NCT04866290) has been completed. Another Phase III prospective randomized controlled study for HCC (NCT01387932) compared the efficacy of DOX‐loaded HepaSphere microspheres with conventional TACE. These studies provide further clinical evidence for the application of HepaSphere in specific indications and dosing regimens. DC Bead‐LUR is another microsphere carrier capable of loading the chemotherapeutic drug lobaplatin. After entering the tumor vasculature, it slowly releases the drug, enabling local sustained delivery [404]. Research on DC Bead‐LUR (lobaplatin‐loaded drug‐eluting microspheres) for treating colorectal cancer liver metastases, among other conditions, had reached Phase II clinical trials (NCT02525380). Several clinical studies have evaluated it. For example, a prospective registry study aimed at assessing the safety and efficacy of irinotecan‐loaded drug‐eluting microspheres for colorectal cancer liver metastases (NCT04866290) has been completed [405].

In summary, leveraging their unique three‐dimensional network structure, hydrogel systems have enabled diverse applications in oncology, ranging from local bladder instillation and intraoperative immune activation to vascular interventional embolization. Currently, certain technological pathways, such as local instillation and vascular embolization, have yielded mature products successfully applied in clinical practice. In contrast, novel intraoperative hydrogels designed to modulate the TME and elicit systemic immunity represent cutting‐edge exploratory directions, most of which are still in early‐stage clinical research. Despite the considerable promise demonstrated by hydrogels in preclinical studies of cancer immunotherapy, their clinical translation faces systemic challenges spanning from fabrication to practical application. Due to the complex structural characteristics of hydrogels, they are highly sensitive to synthesis parameters, which directly affect drug release kinetics and therapeutic consistency. Therefore, the main obstacle lies in achieving large‐scale repeatable production under good manufacturing practice standards [406, 407]. Furthermore, the regulatory framework for such innovative combination products remains underdeveloped, and their unique localized, sustained‐release mode of action necessitates novel approaches for evaluating efficacy and safety beyond conventional paradigms. Additionally, practical challenges in clinical application cannot be overlooked. The injectability of hydrogels must strike a precise balance between flowability and in situ gelation stability to ensure accurate delivery and prolonged retention of therapeutics at the target site [408, 409]. With advancing integration of materials science, pharmaceutical engineering, and clinical medicine, hydrogel systems are poised to play an increasingly pivotal role in the era of precision medicine.

## 5 Conclusion and Future Perspectives

In recent years, hydrogel formulations have demonstrated broad application prospects in the field of cancer immunotherapy. As a class of polymeric materials featuring a three‐dimensional network structure, hydrogels provide innovative solutions for cancer immunotherapy by virtue of their excellent biocompatibility, tunable physicochemical properties, and versatile functional design [10, 256, 410]. This review discusses the research progress and future outlook of hydrogel platforms in cancer immunotherapy, focusing on their construction strategies, immunological mechanisms, clinical advancements, and existing challenges. Significant progress has been made in drug delivery, regulation of the immune microenvironment, and combination therapy, with particularly notable performance in achieving controlled drug release and ameliorating the immunosuppressive TME. These advances offer new avenues to overcome the limitations of conventional immunotherapies, such as restricted efficacy and significant toxic side effects.

Furthermore, hydrogel technology exhibits multifaceted potential in precision oncology. First, in terms of treatment localization, the design of temperature‐, pH‐, or enzyme‐responsive hydrogels enables the targeted release of therapeutics at the tumor site. Second, for precise immune regulation, hydrogels can serve as efficient carriers for cytokines, immune checkpoint inhibitors, and other agents, allowing for targeted intervention in specific immune pathways. Moreover, in combined therapy strategies, hydrogels can codeliver chemotherapeutic agents, immunomodulators, and cellular therapeutics to achieve multimodal synergistic treatment. Finally, in personalized medicine, hydrogel compositions can be tailored based on the specific molecular profile of a patient's tumor, paving the way for truly precision medicine.

However, the clinical translation of hydrogels still faces multiple challenges. The primary issue involves matching material biosafety with the required duration of immune response; excessively rapid degradation can terminate therapy prematurely, while overly slow degradation may provoke chronic inflammation. Additionally, scaling up from laboratory to clinical production presents bottlenecks, including complex manufacturing processes, high costs, and difficulties in ensuring batch‐to‐batch consistency. Another core challenge lies in quantitatively predicting hydrogel behavior within the individualized TME to achieve genuinely precise regulation [411, 412].

To address these challenges, future breakthroughs should focus on developing a new generation of smart biomaterials. Priority should be given to resolving the critical mismatch between hydrogel degradation kinetics and the required duration of immunotherapy, as this directly impacts therapeutic efficacy and safety. Efforts must be dedicated to designing responsive hydrogels that can intelligently adjust their degradation rates in accordance with dynamic changes in the in vivo microenvironment, while also exploring novel biomaterials with intrinsic immunomodulatory functions. Through the deep integration of materials science, immunology, and clinical medicine, the goal is to construct integrated theranostic platforms capable of both precise delivery and real‐time monitoring, thereby accelerating the clinical translation of hydrogel technology in cancer immunotherapy.

## Author Contributions

X. H. C., S. W., Y. Y. Z., L. F., Y. C., and X. Z. Y. drafted this manuscript and prepared the figures. K. M., J. Z., and M. L. designed the framework for the entire manuscript, provided detailed guidance, and financial support. All authors have checked the manuscript and agree to be publication.

## Funding

This work is supported by the Regional Innovation and Development Joint Fund Key Project of the National Natural Science Foundation of China (U24A20735), the National Natural Science Foundation of China (82473289), and the Key Research Program of Science and Technology Department of Sichuan (23NSFJQ0104).

## Ethics Statement

The authors have nothing to report.

## Conflicts of Interest

The authors declare no conflicts of interest.

## Data Availability

Data sharing is not applicable to this article as no new data were created or analyzed in this study.
